# Profiling estrogen, progesterone, and androgen receptors in colorectal cancer in relation to gender, menopausal status, clinical stage, and tumour sidedness

**DOI:** 10.3389/fendo.2023.1187259

**Published:** 2023-05-03

**Authors:** Bassem Refaat, Akhmed Aslam, Shakir Idris, Ahmed H. Almalki, Mofareh Y. Alkhaldi, Hassan A. Asiri, Riyad A. Almaimani, Abdulrahman Mujalli, Faisal Minshawi, Sara A. Alamri, Mona I. AlHussain, Badee A. Baltow, Mansour H. Alqasmi, Ghaiyda T. Basfar, Ohoud M. Alosaimi, Ibrahim A. Muhayya

**Affiliations:** ^1^ Laboratory Medicine Department, Faculty of Applied Medical Sciences, Umm Al-Qura University, Makkah, Saudi Arabia; ^2^ Regional Laboratory and Central Blood Bank, Ministry of Health, Jizan, Saudi Arabia; ^3^ Laboratory And Blood Bank Department, Asir Central Hospital, Abha, Saudi Arabia; ^4^ Forensic Medicine Department, Health Affairs General Directorate in Assir, Abha, Saudi Arabia; ^5^ Biochemistry Department, Faculty of Medicine, Umm Al-Qura University, Makkah, Saudi Arabia; ^6^ Histopathology Department, King Abdullah Medical City, Makkah, Saudi Arabia; ^7^ Clinical Laboratories, Al-Noor Specialist Hospital, Makkah, Saudi Arabia; ^8^ Clinical Laboratories, Eradah and Mental Health Complex, Ministry of Health, Taif, Saudi Arabia

**Keywords:** testosterone, cell cycle, apoptosis, left-right dichotomy, mifepristone, bicalutamide, estrogen receptor-β (ERβ), estrogen receptor-α (ERα)

## Abstract

**Background:**

Although estrogen (ERα/ERβ), progesterone (PGR), and androgen (AR) receptors are pathologically altered in colorectal cancer (CRC), their simultaneous expression within the same cohort of patients was not previously measured.

**Methods:**

ERα/ERβ/PGR/AR proteins were measured in archived paired normal and malignant colon specimens (n =120 patients) by immunohistochemistry, and results were analyzed by gender, age (≤50 vs. ≥60 years), clinical stages (early-stage I/II vs. late-stage III/IV), and anatomical location (right; RSCs vs. left; LSCs). Effects of 17β-estradiol (E2), progesterone (P4), and testosterone alone or combined with the specific blockers of ERα (MPP dihydrochloride), ERβ (PHTPP), PGR (mifepristone), and AR (bicalutamide) on cell cycle and apoptosis were also measured in the SW480 male and HT29 female CRC cell lines.

**Results:**

ERα and AR proteins increased, whilst ERβ and PGR declined markedly in malignant specimens. Moreover, male neoplastic tissues showed highest AR expression, whilst ERβ and PGR weakest alongside ERα strongest expression was seen in cancerous tissues from women aged ≥60 years. Late-stage neoplasms also revealed maximal alterations in the expression of sex steroid receptors. By tumor location, LSCs disclosed significant elevations in ERα with marked declines in PGR compared with RSCs, and ERα strongest alongside PGR weakest expression was detected in advanced LSCs from women aged ≥60 years. Late-stage LSCs from females aged ≥60 years also showed weakest ERβ and strongest AR expression. In contrast, male RSC and LSC tissues exhibited equal ERβ and AR expression in all clinical stages. ERα and AR proteins also correlated positively, whereas ERβ and PGR inversely, with tumor characteristics. Concomitantly, E2 and P4 monotherapies triggered cell cycle arrest and apoptosis in the SW480 and HT29 cells, and while pre-treatment with ERα-blocker enhanced the effects of E2, ERβ-blocker and PGR-blocker suppressed the E2 and P4 anti-cancer actions, respectively. In contrast, treatment with the AR-blocker induced apoptosis, whilst co-treatment with testosterone hindered the effects.

**Conclusions:**

This study advocates that protein expression of sex steroid receptors in malignant tissues could represent prognostic markers, as well as hormonal therapy could provide an alternative strategy against CRC, and their efficacies could be dependent on gender, clinical stage, and tumor location.

## 1 Introduction

Worldwide, colorectal cancer (CRC) is the most prevalent and fatal gastrointestinal neoplastic disease (1, 2). Several factors increase the risk of CRC, among which tumor location has recently been shown to correlate with tumor characteristics and clinical outcomes. In detail, right-sided cancers (RSCs) are linked with larger tumor size, poor differentiation, mucinous histology, distant metastasis, and worse prognosis compared to left-sided cancers (LSCs) (3–5). Moreover, epidemiological observations have consistently shown lower risk of developing CRC in pre-menopausal women, whilst post-menopausal women using hormonal replacement therapy (HRT) also had markedly lower incidence of CRC relative to nonusers, as well as age-matched men (6–9). Moreover, better prognosis has been noted in CRC female patients aged 18-44 years relative to women > 50 years of age, as well as men of the same age (10). Therefore, it has been suggested that sex steroid hormones could contribute to colon oncogenesis (6–10).

Although the production of sex steroid hormones mainly occurs in the gonads, other peripheral tissues, including colon, express the enzymes required for the biogenesis of sex hormones, including progesterone (P4), testosterone, and the most potent estrogen, 17β-estradiol (E2) (11–13). Colonic mucosa could also respond to sex hormones, since estrogen (ERα & ERβ) (14–16), progesterone (PGR) (17–19), and androgen (AR) (20–22) receptors were detected and showed gender-dependent expression profiles. Moreover, ERβ, PGR and AR in normal colonic tissue are abundant, whilst ERα is weakly expressed (14–22). However, the protein expression of ERα (23–25) and AR (26) increases, whereas ERβ (27–29) and PGR (23, 30–32) decline, markedly in cancerous colonic tissues, and they correlated with the tumor clinicopathological characteristics and/or prognosis. Experimental studies have also shown that E2 induced ERβ-mediated anti-cancer actions, whilst promoting oncogenic effects through ERα in colonic cells, both *in vivo* and *in vitro* (33–36). Moreover, treatment with P4 induced apoptosis and inhibited cancer progression (30, 36), whereas testosterone therapy triggered cell growth and colon carcinogenesis (37–40), *in vitro* and in animal models. Hence, the authors suggested that E2, through ERβ (33–36), and P4 *via* PGR (30, 36) could act as tumor suppressors, whilst activation of ERα by E2 (33–36) and AR by testosterone (37–40) could promote the development and progression of colon neoplasia.

However, none of the earlier studies measured the expression of all the sex steroid hormone receptors within the same cohort of clinical samples or analyzed the results in relation to menopausal status in women. Moreover, little is currently known about the expression of sex steroid receptors with respect to tumor sidedness. Hence, this study investigated the protein expression of ERα, ERβ, PGR, and AR in paired non-cancerous and cancerous tissues collected from patients diagnosed with CRC, and the results were analyzed according to gender, age, clinical stage, and tumor anatomical sites. To support the clinical findings, the SW480 male and HT29 female CRC cell lines were also treated with E2, P4, and testosterone alone or combined with their specific nuclear receptor blockers to measure their effects on cell cycle and apoptosis according to gender. Understanding the roles of sex steroid hormones in colon oncogenesis could provide better prognostic markers and/or alternative hormonal therapies for CRC.

## 2 Materials and methods

### 2.1 Clinical study and sample collection

Paired Formalin-Fixed Paraffin-Embedded (FFPE) malignant and their corresponding non-malignant colonic specimens were collected from the archives of Histopathology Department of King Abdullah Medical City in Makkah (KAMC) following ethical approval (#19-498). The study included 120 Saudi male and female patients between January 2019 and December 2021, aged between ≥ 18 years and ≤ 50 years, or ≥ 60 years and who were diagnosed with primary sporadic CRC. Moreover, all patients did not receive neoadjuvant chemo/radiotherapy prior to their surgery. Patients with a history of inherited or recurrent CRC and/or aged between 51 and 59 years old were excluded to ensure menopausal status.

The final diagnosis with the histopathological staging were based on institutional clinical management guidelines that followed the 8^th^ edition of the American Joint Committee on Cancer tumor-node-metastasis (TNM) staging system. By retrieving the pathology and surgical reports, tumors located from the cecum to the margin of hepatic flexure were categorized as right-sided cancer (RSC), whilst neoplasms located from the splenic flexure to the rectum were considered left-sided cancer (LSC) (4). All the retrieved tissue blocks were examined by consultant histopathologists in KAMC to ensure adequacy.

#### 2.1.1 Immunohistochemistry

Primary mouse monoclonal IgG antibodies (Santa-Cruz Biotechnology Inc.; CA, USA) were used to detect ERα (#sc-8002), ERβ (#sc-53494), PGR (#sc-810), and AR (#sc-7305) in 5-μm sections from each non-malignant and cancerous tissues. Endogenous peroxidases were blocked by using a BLOXALL® Solution (#SP-6000-100; Vector Laboratories Inc., CA, USA) for 15 min. Subsequently, the sections were incubated overnight with the primary antibodies (1:200 concentration for all) at 4°C. After washing, the sections were treated with ImmPRESS® HRP Horse Anti-mouse (#MP-7402) IgG Plus Polymer Peroxidase Kits, as per the manufacturer’s protocol (Vector Laboratories Inc.). The same protocol was also used with the negative control sections, but primary isotype mouse (#sc-2025) IgG antibodies (Santa-Cruz Biotechnology Inc.) were used to control for non-specific staining. The sections were studied on a Leica DMi8 microscope (Leica Microsystems, Wetzlar, Germany) and images were acquired from 10 random fields/section with a 20× objective.

The ImageJ software (https://imagej.nih.gov/ij/) was used to measure the protein expression by the IHC Image Analysis Toolbox, as reported elsewhere (4, 41). Briefly, the stained areas (ROI) were identified, and the stain intensity with the percentage of stained areas were measured. The IHC scores were calculated by the following equation, as previously described equation (4, 41):


$$\begin{array}{c} IHC\;stain\;intensity\;\\ =\;[\left(255\;-\;ROI\;stain\;score\right)\\ \;\times \;\%\;ROI\;\left(ROI\;pixels/total\;image\;pixels\;\times \;100\right)] \end{array}$$

The IHC scores for each receptor were then compared between the paired normal and cancerous tissues of each patient (normal *vs.* malignant), as well as between the clinical stages (early [I/II] *vs.* late [III/IV]), both genders (male *vs.* female), tumor sites (RSC *vs.* LSC), and age groups (≤ 50 *vs.* ≥ 60 years).

### 2.2 *In vitro* experiments

#### 2.2.1 Chemicals and reagents

Ultrapure (>99%) testosterone hormone (#86500) was from Sigma-Aldrich Co. (MO, USA), whilst 17β-Estradiol (E2; #HY-B0141) and progesterone (P4; #HY-N0437) hormones, alongside the specific receptor blockers of ERα (MPP dihydrochloride; #HY-103454), ERβ (PHTPP; #HY-103456), PGR (mifepristone; #HY-13683), and AR (bicalutamide; #HY-14249) were obtained from MedChemExpress LLC (Princeton, NJ, USA). Cell culture media (DMEM & RPMI-1640), fetal bovine serum (FBS), antibiotic-antimycotic solution, and sterile 96-well and 6-well plates were from Thermo Fisher Scientific (MT, USA). The female (HT29) and male (SW480) human colon cancer cell lines were from the American Type Culture Collection (VA, USA).

#### 2.2.2 Cell culture and cell viability assay

The HT29 cells were cultured in RPMI-1640, whilst DMEM was used for the SW480, and all media included 10% FBS and 1% antibiotic-antimycotic solution. All cells were sub-cultured, and growth maintained at 37°C in a humified incubator with 5% CO_2_. Following seeding in 96-well plates, both cell lines were treated with each drug alone for 48h to determine the concentrations associated with 50% inhibition (IC50) or 10% increase (EC10) of cell viability using the MTT cytotoxicity assay, as described earlier (42, 43).

#### 2.2.3 Cell cycle analysis

Following determination of IC50 or EC10 concentrations, the cells were seeded in 6-well plates and divided into the following groups: untreated control (CT), E2 (E2), P4 (P4), and testosterone (T) single treatments, alongside the MPP + E2 (E/α), PHTPP + E2 (E/β), mifepristone + P4 (P/M), and bicalutamide + testosterone (T/B) dual therapies. Each receptor blocker was added for a total duration of 48h in the co-therapy protocols, whilst E2, P4, or testosterone therapies were incubated for 24h and were initiated in the single and dual treatment groups 24h after adding their corresponding receptor blockers for 24h.

At the end of experiments, the SW480 and HT29 cells were trypsinised and suspended, washed twice with PBS, and fixed in ice-cold 70% ethanol for 24h at 4°C. Cells were then treated with RNase A (20 μg/ml; Thermo Fisher) for 15 min after washing twice in PBS. Propidium iodide (PI; 2 µg/ml; Thermo Fisher) was used to stain cellular DNA immediately prior to cell cycle analysis with an Acea Novocyte 3000 flow cytometer (Agilent Technologies, CA, USA). The percentage of cells in each phase of cell cycle were determined for 20,000 events (n = 3/group) by the NovoExpress software cell cycle algorithm, as reported earlier (42, 43).

#### 2.2.4 Apoptosis assay

The Annexin V-FITC/PI Apoptosis Assay Kit (Thermo Fisher Scientific) was used to measure the effects of the different therapies on cell death. Briefly, the SW480 and HT29 cells were washed twice with ice cold PBS following the different treatments and re-suspended in 100 μl of 1× Annexin V (AV) binding buffer. Cell suspensions were then incubated in the dark for 15 min at room temperature, after the addition of AV-FITC (5 μl) and PI (1 μl). Before analysing with the Acea Novocyte 3000 flow cytometer, the samples were kept on ice after adding 400 μl of AV binding buffer. The percentages of live (non-stained), early (AV+/PI-) and late apoptotic (AV+/PI+), and dead (AV-/PI+) cells are shown as mean ± SD (n = 3).

### 2.3 Statistical analysis

SPSS statistical analysis software version 25 was used for data analysis. Normality and homogeneity of all continuous variables were determined by the Kolmogorov and Smirnov’s test and the Levene test, respectively. Ordinal and discontinuous variables are shown as numbers with percentages, and Chi-square (χ^2^) test following cross-tabulation were used to measure frequencies. While Student’s t or Mann-Whitney U tests were applied to compare between two groups based on normality, one-way analysis of variance (ANOVA) with Tukey’s HSD or Games-Howell *post-hoc* tests were used for comparing between several groups based on equality of variance. Continuous variables are shown as mean ± standard deviation (SD) or median with interquartile range (IQR; 25^th^ – 75^th^ percentiles), according to data normality. Correlations were measured by Pearson’s or Spearman’s tests based on data normality. Significance was considered with P< 0.05.

## 3 Results

### 3.1 Clinicopathological characteristics of colonic tumors

Overall, the patients included 64 males (53.3%) and 56 females (46.7%), and the mean of age was comparable between both genders (58.4 ± 13.2 and 58.7 ± 13.1 years, respectively). The T stages were T1 in two (1.7%), T2 in 13 (10.8%), T3 in 72 (60%) and T4 in 33 (27.5%) patients. Regional lymph nodes were positive for malignant cells in 63 (52.5%) patients, whilst distant liver metastasis (stage M1a) was detected in 16 patients (13.3%). The most prevalent histology was Adenocarcinoma (n = 92; 76.7%) whilst the remainder was mucinous carcinoma. Additionally, 20 (16.7%), 72 (60%) and 28 (23.3%) patients had poorly, moderately, and well-differentiated cancers, respectively. Moreover, 42 (35%) and 28 (23.3%) patients were positive for lymphovascular and perineural invasions, respectively. AS per the TNM staging criteria, 50 (41.7%) cases were clinically diagnosed as early (stages I/II) and the remainder (58.3%) as advanced (stages III/IV) malignancies.

According to tumor anatomical sites, RSCs were less frequent (n = 41; 34.2%) and associated with markedly higher rates of mucinous neoplasms, poor differentiation, and distant metastasis relative to LSCs (**Supplementary Table 1**). However, both the RSC and LSC groups showed comparable average age, as well as distributions of genders, T and N stages, lymphovascular and perineural invasions, and rates of early (I/II) and late (III/IV) cancer stages (**Supplementary Table 1**). By classifying the patients according to gender and age groups, there were 22 men (18.3%) and 20 women (16.7%) aged ≤ 50 years, whilst patients aged ≥ 60 years included 42 males (35%) and 36 females (30%). All the clinicopathological features were similar between the different groups, except for the rate of T4 stage that was markedly higher in male patients (**Table 1**). 3.2 Protein expression of sex steroid receptors in clinical samples by IHC.

**Table 1:** The clinicopathological characteristics of CRC according to patients’ gender and age groups (n = 120).

	Male patients (n = 64; 53.3%)		Female patients (n = 56; 46.7%)		P-value
	≤ 50 years (n = 22; 18.3%)	≥ 60 Years (n = 42; 35%)	≤ 50 years (n = 20; 16.7%)	≥ 60 Years (n = 36; 30%)	
Tumor sidedness					
Right-sided	8 (6.6%)	13 (10.8%)	7 (5.9%)	13 (10.8%)	0.9
Left-sided	14 (11.7%)	29 (24.2%)	13 (10.8%)	23 (19.2%)	
Tumor infiltration (T stage)					
T1	0 (0%)	0 (0%)	2 (1.7%)	0 (0%)	**0.04**
T2	2 (1.7%)	8 (6.6%)	2 (1.7%)	1 (0.8%)	
T3	12 (10%)	23 (19.2%)	11 (9.1%)	26 (21.7%)	
T4	8 (6.6%)	11 (9.2%)	5 (4.2%)	9 (7.5%)	
*Median (IQR) of tumor volume (cm^3^)*	11.5 (4.8 – 27.5)	8.4 (4.4 – 15.3)	9.5 (4.5 – 18.8)	10.5 (5.5 – 29.5)	0.5
Lymph node (N stage)					
N0	9 (7.5%)	22 (18.4%)	12 (10%)	14 (11.7%)	0.7
N1	8 (6.6%)	11 (9.1%)	5 (4.2%)	14 (11.7%)	
N2	5 (4.2%)	9 (7.5%)	3 (2.5%)	8 (6.6%)	
Distant metastasis (M stage)					
M0	19 (15.8%)	38 (31.7%)	17 (14.2%)	30 (25%)	0.8
M1	3 (2.5%)	4 (3.3%)	3 (2.5%)	6 (5%)	
Histology					
Adenocarcinoma	18 (15%)	33 (27.5%)	16 (13.4%)	25 (20.9%)	0.6
Mucinous	4 (3.3%)	9 (7.5%)	4 (3.3%)	11 (9.1%)	
Differentiation					
Poor	3 (2.5%)	4 (3.3%)	4 (3.3%)	9 (7.5%)	0.5
Moderate	12 (10%)	28 (23.4%)	11 (9.1%)	21 (17.5%)	
Well	7 (5.8%)	10 (8.3%)	5 (4.2%)	6 (5%)	
Lymphovascular invasion					
No	12 (10%)	28 (23.4%)	16 (13.4%)	22 (18.3%)	0.3
Yes	10 (8.3%)	14 (11.7%)	4 (3.3%)	14 (11.7%)	
Perineural invasion					
No	14 (11.7%)	32 (26.7%)	16 (13.4%)	30 (25%)	0.2
Yes	8 (6.6%)	10 (8.3%)	4 (3.3%)	6 (5%)	
AJCC TNM stages					
Stages I/II	8 (6.6%)	19 (15.8%)	12 (10%)	11 (9.1%)	0.1
Stages III/IV	14 (11.7%)	23 (19.2%)	8 (6.6%)	25 (20.9%)	

Bold values indicates statistical significance.

#### 3.2.1 Estrogen receptors

In non-malignant tissues, the antibodies against ERα (**Supplementary Figure 1**) and ERβ (**Supplementary Figure 2**) labelled the cytoplasm and nuclei of colonic epithelia, and the immunostaining of the latter was substantially stronger. Moreover, the expression of ERα was markedly higher in the female right and left non-cancerous tissues compared with male patients (**Supplementary Figure 1**; P< 0.01 for both). While ERα was equal between the male non-malignant specimens during the different cancer stages, women ≥ 60 years showed significantly higher IHC scores in the left-sided non-neoplastic tissues obtained from the early and late stages relative to women aged ≤ 50 years (**Supplementary Figure 1**; P< 0.001). On the other hand, female patients aged ≤ 50 years disclosed the highest ERβ expression in the right and left non-malignant specimens compared with both age groups in males, as well as females ≥ 60 years of age (**Supplementary Figure 2**, P< 0.001 for all). Although ERβ expression in the male non-cancerous tissues was also equal between both age groups, the non-cancerous tissues from the late stages of RSCs and LSCs had markedly lower IHC scores compared with their counterpart early stages (**Supplementary Figure 2**; P< 0.01 for both). Furthermore, the non-malignant tissues from women aged ≥ 60 years revealed the lowest ERβ protein expression compared with all groups, and the lowest scores were detected in samples obtained from late RSCs (**Supplementary Figure 2**).

**Figure 1:**
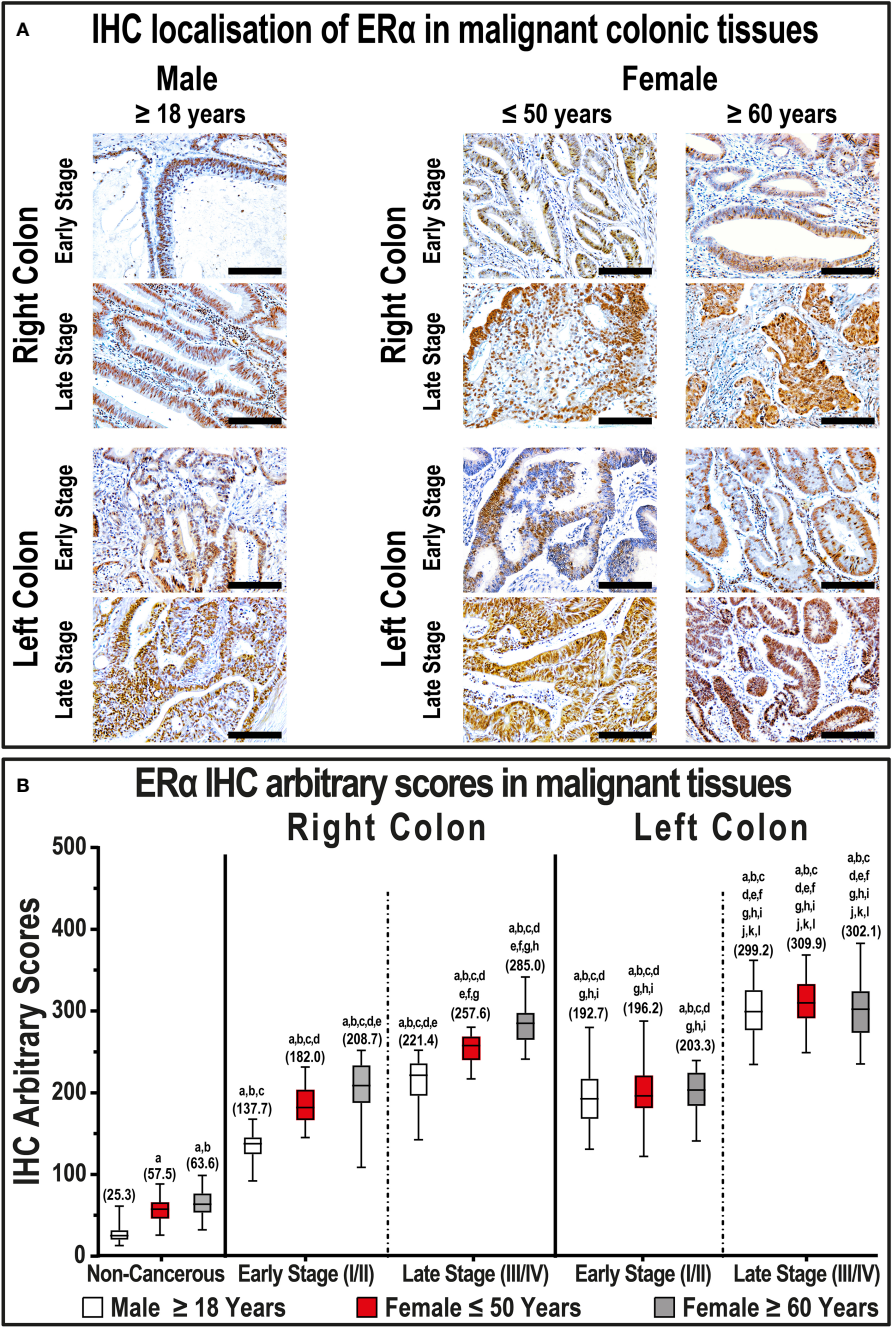
**(A)** Immunohistochemical localization of ERα in malignant colonic tissues (n = 120 patients; 20× objective; Scale bar = 15 μm) alongside **(B)** its IHC arbitrary scores are shown as boxplots according to gender, age, tumor sides and cancer stages. (a = P< 0.05 compared with normal specimens from males ≥ 18 years; b = P< 0.05 compared with normal specimens from females ≤ 50 years; c = P< 0.05 compared with normal specimens from females ≥ 60 years; d = P< 0.05 compared with early-stage right-sided malignant samples from males ≥ 18 years; e = P< 0.05 compared with early-stage right-sided malignant samples from females ≤ 50 years; f = P< 0.05 compared with early-stage right-sided malignant samples from females ≥ 60 years; g = P< 0.05 compared with late-stage right-sided malignant samples from males ≥ 18 years; h = P< 0.05 compared with late-stage right-sided malignant samples from females ≤ 50 years; i = P< 0.05 compared with late-stage right-sided malignant samples from females ≥ 60; j = P< 0.05 compared with early-stage left-sided malignant samples from males ≥ 18 years; k = P< 0.05 compared with early-stage left-sided malignant samples from females ≤ 50 years; l = P< 0.05 compared with early-stage left-sided malignant samples from females ≥ 60 years; m = P< 0.05 compared with late-stage left-sided malignant samples from males ≥ 18 years; n = P< 0.05 compared with late-stage left-sided malignant samples from females ≤ 50 years).

In general, the protein expression of ERα increased significantly (213.4; IQR: 192.9 – 286.3) in cancerous compared with non-cancerous tissues (36.9; IQR: 24.1 – 63.2; P< 0.0001). Moreover, left-side cancers (257.9; IQR: 200.3 – 307.1) had higher ERα expression relative to RSCs (222.5; IQR: 183.3 – 248.7; P< 0.0001). According to clinical stage, late-stage right and left cancers showed markedly higher IHC scores relative to their corresponding early-stage cancerous tissues, and the highest scores were detected in late-stage LSCs (**Figure 1**; P< 0.0001). However, the ERα IHC scores in late-stage LSCs were comparable between both genders, whilst women aged ≥ 60 years and diagnosed with advanced RSCs displayed the strongest immunostaining relative to men, as well as females aged ≤ 50 years, diagnosed with early-stage RSCs (**Figure 1**; P< 0.001 for both).

**Figure 2:**
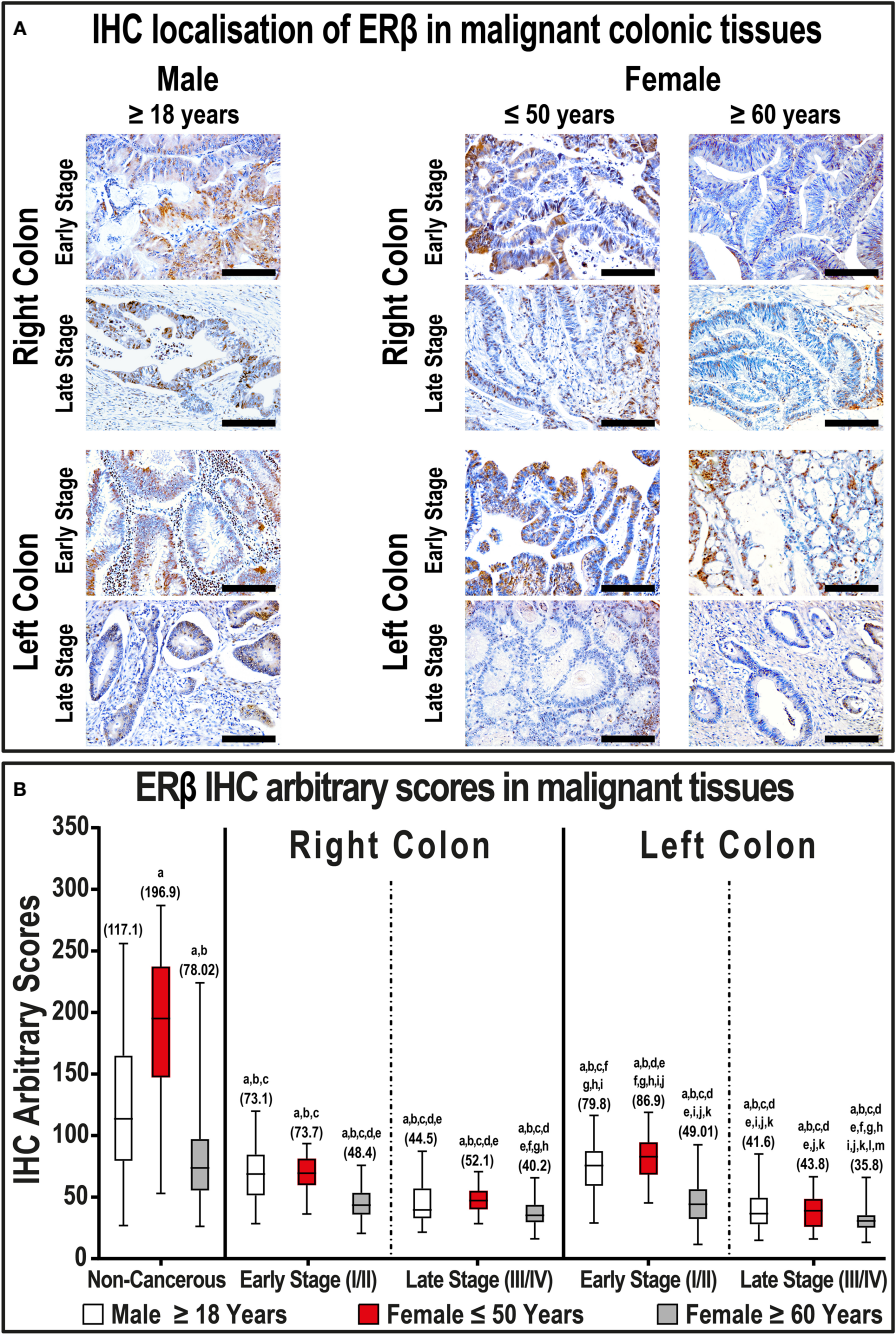
**(A)** Immunohistochemical localization of ERβ in malignant colonic tissues (n = 120 patients; 20× objective; Scale bar = 15 μm) alongside **(B)** its IHC arbitrary scores are shown as boxplots according to gender, age, tumor sides and cancer stages. (a = P< 0.05 compared with normal specimens from males ≥ 18 years; b = P< 0.05 compared with normal specimens from females ≤ 50 years; c = P< 0.05 compared with normal specimens from females ≥ 60 years; d = P< 0.05 compared with early-stage right-sided malignant samples from males ≥ 18 years; e = P< 0.05 compared with early-stage right-sided malignant samples from females ≤ 50 years; f = P< 0.05 compared with early-stage right-sided malignant samples from females ≥ 60 years; g = P< 0.05 compared with late-stage right-sided malignant samples from males ≥ 18 years; h = P< 0.05 compared with late-stage right-sided malignant samples from females ≤ 50 years; i = P< 0.05 compared with late-stage right-sided malignant samples from females ≥ 60; j = P< 0.05 compared with early-stage left-sided malignant samples from males ≥ 18 years; k = P< 0.05 compared with early-stage left-sided malignant samples from females ≤ 50 years; l = P< 0.05 compared with early-stage left-sided malignant samples from females ≥ 60 years; m = P< 0.05 compared with late-stage left-sided malignant samples from males ≥ 18 years; n = P< 0.05 compared with late-stage left-sided malignant samples from females ≤ 50 years).

In contrast, ERβ protein declined in cancerous specimens (51.3; IQR: 40.4 – 72.1) compared with their corresponding non-malignant colonic tissues (114.1; IQR: 76.5 – 174.0; P< 0.001; **Figure 2**). Furthermore, the protein expression of ERβ was equal between right (49.4; IQR: 39.8 – 66.2) and left-sided (50.9; IQR: 36.2 – 75.2) tumors. However, ERβ immunostaining decreased with cancer progression in both genders, and the lowest IHC scores were observed in late-stage RSCs and LSCs relative to the early-stage specimens (**Figure 2**, P< 0.01 for all). By further analysis, right and left-sided malignant tissues obtained from women ≥ 60 years of age showed significantly lower ERβ expression than women aged ≤ 50 years, as well as men, and the lowest IHC scores were detected in the late stages of LSCs (**Figure 2**).

#### 3.2.2 Progesterone receptor

The immunostaining of PGR was visualized in the cytoplasm and nuclei of non-neoplastic colonic specimens from both genders and demonstrated moderate to strong intensities (**Supplementary Figure 3**). The expression of PGR was also significantly higher in the left-sided compared with right-sided non-cancerous tissues obtained from both genders (P< 0.01 for both). Moreover, the strongest PGR expression was observed in right and left non-malignant specimens obtained from females aged ≤ 50 years compared with the different male age groups and women ≥ 60 years (**Supplementary Figure 3**). Although the expression of PGR in the right-sided non-cancerous samples was equal in females ≥ 60 years and both male age groups, the IHC scores were significantly lower in the former group in the left-sided samples (**Supplementary Figure 3**).

**Figure 3:**
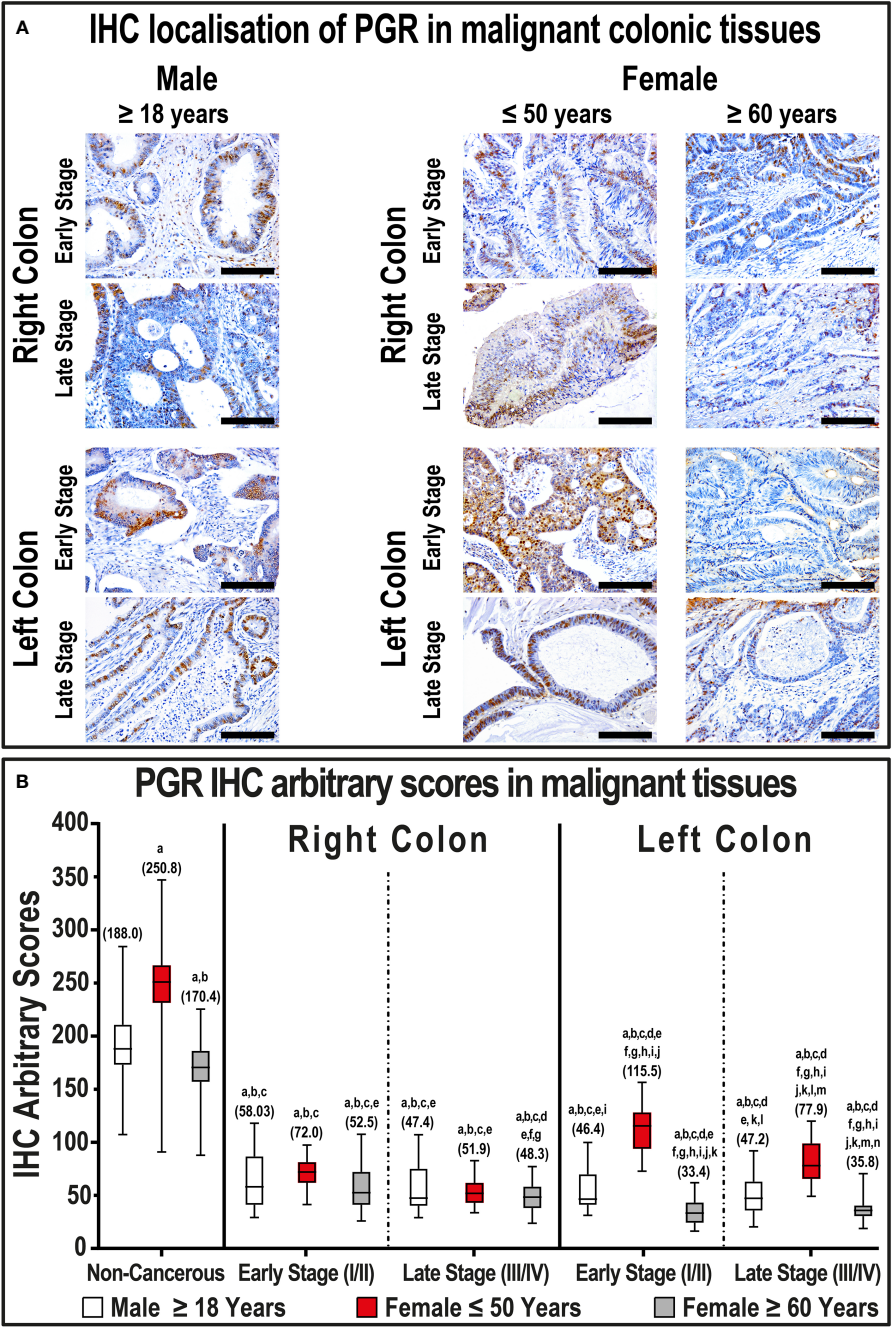
**(A)** Immunohistochemical localization of PGR in malignant colonic tissues (n = 120 patients; 20× objective; Scale bar = 15 μm) alongside **(B)** its IHC arbitrary scores are shown as boxplots according to gender, age, tumor sides and cancer stages. (a = P< 0.05 compared with normal specimens from males ≥ 18 years; b = P< 0.05 compared with normal specimens from females ≤ 50 years; c = P< 0.05 compared with normal specimens from females ≥ 60 years; d = P< 0.05 compared with early-stage right-sided malignant samples from males ≥ 18 years; e = P< 0.05 compared with early-stage right-sided malignant samples from females ≤ 50 years; f = P< 0.05 compared with early-stage right-sided malignant samples from females ≥ 60 years; g = P< 0.05 compared with late-stage right-sided malignant samples from males ≥ 18 years; h = P< 0.05 compared with late-stage right-sided malignant samples from females ≤ 50 years; i = P< 0.05 compared with late-stage right-sided malignant samples from females ≥ 60; j = P< 0.05 compared with early-stage left-sided malignant samples from males ≥ 18 years; k = P< 0.05 compared with early-stage left-sided malignant samples from females ≤ 50 years; l = P< 0.05 compared with early-stage left-sided malignant samples from females ≥ 60 years; m = P< 0.05 compared with late-stage left-sided malignant samples from males ≥ 18 years; n = P< 0.05 compared with late-stage left-sided malignant samples from females ≤ 50 years).

Generally, the expression of PGR protein declined markedly in malignant tissues (53.0; IQR: 43.2 – 61.8) relative to their corresponding non-malignant colonic samples (187.3; IQR: 169.7 – 216.0; P< 0.001). Left-sided tumors (45.9; IQR: 35.9 – 60.2) also showed markedly lower PGR expression relative to right-sided lesions (62.3; IQR: 51.1 – 81.1; P< 0.001). Furthermore, PGR decreased with cancer progression in both genders, and the lowest IHC scores were detected in women ≥ 60 years of age and diagnosed with early and late-stage LSCs compared with all groups (**Figure 3**, P< 0.01 for all). In contrast, early and late-stage malignant tissues from women aged ≤ 50 years with LSCs, but not RSCs, had markedly stronger PGR immunostain relative to male cancerous tissues (**Figure 3**).

#### 3.2.3 Androgen receptor

AR showed cytoplasmic and nuclear localization in the non-neoplastic colonic epithelium, and the stain intensity was gender-dependent (**Supplementary Figure 4**). In more detail, the expression of AR was significantly higher in the right-sided non-tumorous tissues obtained from males diagnosed with early-stage cancer compared with their corresponding female specimens (P< 0.01). Moreover, all non-malignant specimens from women ≤ 50 years of age disclosed the lowest expression of AR compared with all non-cancerous specimens obtained from males, as well as females aged ≥ 60 years (**Supplementary Figure 4**). In contrast, non-malignant colonic tissues from women ≥ 60 years of age showed lower AR expression in early-stage, whilst increased significantly in late-stage, RSCs compared with their corresponding male tissues. Moreover, the AR IHC scores in left-sided non-cancerous tissues were equal between both age groups in men, as well as in females ≥ 60 years during the early and late-stage cancers (**Supplementary Figure 4**).

**Figure 4:**
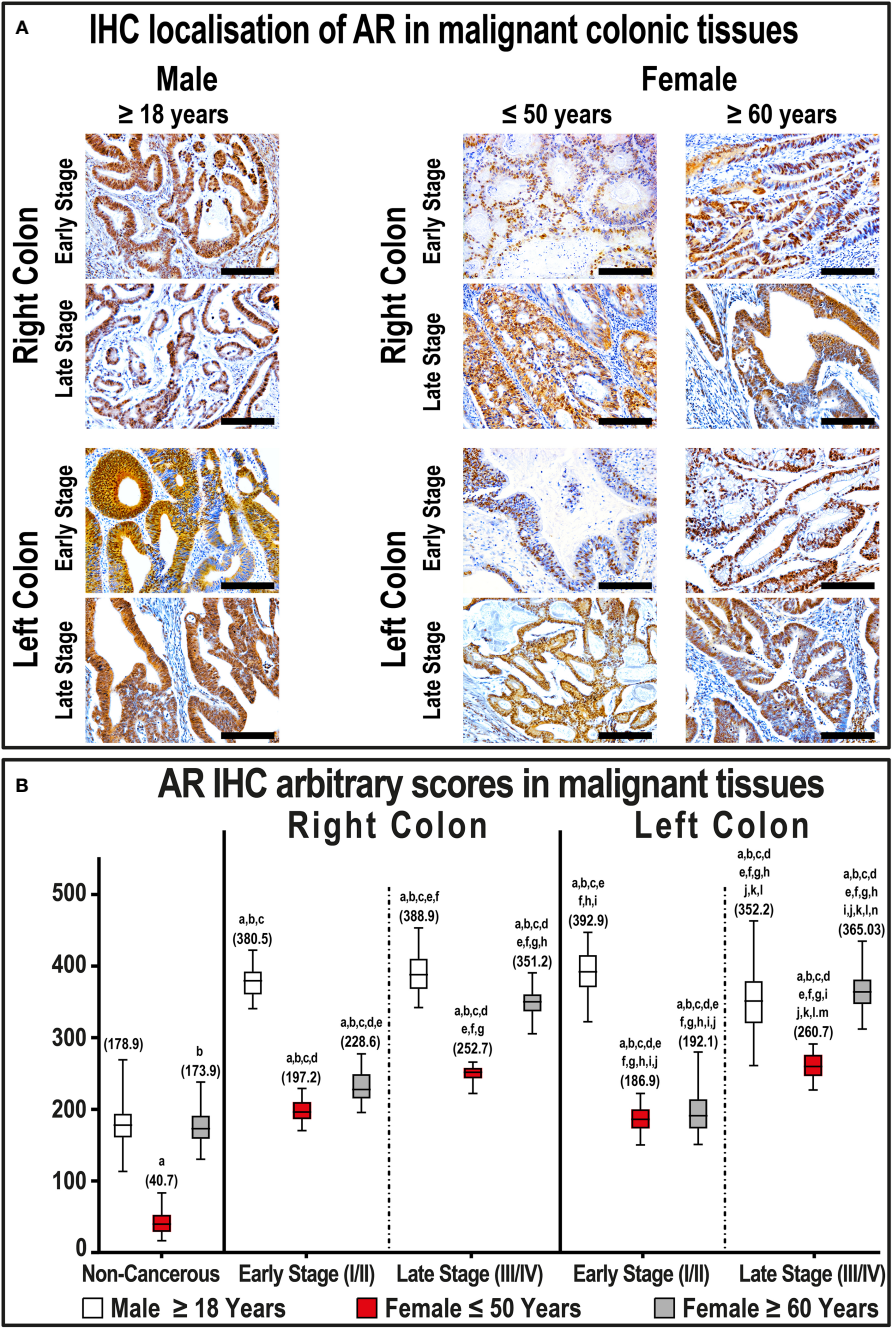
**(A)** Immunohistochemical localization of AR in malignant colonic tissues (n = 120 patients; 20× objective; Scale bar = 15 μm) alongside **(B)** its IHC arbitrary scores are shown as boxplots according to gender, age, tumor sides and cancer stages. (a = P< 0.05 compared with normal specimens from males ≥ 18 years; b = P< 0.05 compared with normal specimens from females ≤ 50 years; c = P< 0.05 compared with normal specimens from females ≥ 60 years; d = P< 0.05 compared with early-stage right-sided malignant samples from males ≥ 18 years; e = P< 0.05 compared with early-stage right-sided malignant samples from females ≤ 50 years; f = P< 0.05 compared with early-stage right-sided malignant samples from females ≥ 60 years; g = P< 0.05 compared with late-stage right-sided malignant samples from males ≥ 18 years; h = P< 0.05 compared with late-stage right-sided malignant samples from females ≤ 50 years; i = P< 0.05 compared with late-stage right-sided malignant samples from females ≥ 60; j = P< 0.05 compared with early-stage left-sided malignant samples from males ≥ 18 years; k = P< 0.05 compared with early-stage left-sided malignant samples from females ≤ 50 years; l = P< 0.05 compared with early-stage left-sided malignant samples from females ≥ 60 years; m = P< 0.05 compared with late-stage left-sided malignant samples from males ≥ 18 years; n = P< 0.05 compared with late-stage left-sided malignant samples from females ≤ 50 years).

Overall, AR protein expression increased significantly in the cancerous colonic tissues (364.9; IQR: 268.1 – 388.8) compared with non-cancerous samples (171.6; IQR: 150.1 – 190.9; P< 0.0001). However, the expression of AR was equal between proximal (358.3; IQR: 256.4 – 388.3) and distal (357.5; IQR: 279.4 – 389.8) cancers. There were also no significant differences between the male cancerous specimens according to tumor sidedness and clinical stages (**Figure 4**), whereas all female right and left-sided malignant samples showed markedly lower AR expression during the different cancer stages compared with their counterpart male malignant tissues (**Figure 4**). Moreover, the expression of AR was significantly lower in RSC and LSC malignant specimens obtained from women aged ≤ 50 years relative to females aged ≥ 60 years (**Figure 4**).

#### 3.2.4 Correlations between tumor clinicopathological characteristics and protein expression of sex steroid receptors

Overall, the IHC scores of ERα in cancerous specimens correlated significantly and inversely with those of ERβ (r = -0.533; P< 0.0001) and PGR (r = -0.259; P< 0.0001), whilst directly with AR (r = 0.117; P< 0.0001). On the other hand, ERβ and PGR in malignant tissues correlated positively together (r = 0.648; P< 0.001), whereas they associated negatively with AR (r = -0.159 and r = -0.367, respectively; P< 0.0001 for both). Moreover, the protein expression of ERα and AR in malignant tissues showed significant positive correlations, whereas ERβ and PGR correlated negatively, with older age, tumour size, N stage, numbers of positive lymph nodes, and advanced cancer stage (**Table 2**).

**Table 2:** Correlations of tumor clinicopathological characteristics with ERα, ERβ, PGR, and AR protein expression in malignant colonic tissues from all patients (n = 120) by Pearson’s correlation test.

	ERα IHC scores	ERβ IHC scores	PGR IHC scores	AR IHC scores
**Female gender**	0.247^**^	-0.239^**^	-0.161	-0.663^**^
**Age**	0.351^**^	-0.254^**^	-0.291^**^	0.150
**Right-sided cancer**	0.143	0.030	-0.136	-0.038
**T stage**	0.114	-0.230^*^	-0.084	0.189^*^
**Tumor Size**	0.291^**^	-0.326^**^	-0.297^**^	0.105
**N Stage**	0.617^***^	-0.579^**^	-0.208^*^	0.320^**^
**Numbers of positive lymph nodes**	0.604^***^	-0.591^**^	-0.259^**^	0.259^**^
**M Stage**	0.206^*^	-0.295^**^	-0.104	0.177
**Mucinous Carcinoma**	0.057	-0.096	-0.144	-0.013
**Poor differentiation**	0.094	-0.058	0.028	-0.029
**Lymphovascular invasion**	0.170	-0.263^**^	-0.162	0.166
**Perineural invasion**	-0.071	-0.125	-0.092	0.124
**Late-stage cancer**	0.728^***^	-0.720^***^	-0.320^**^	0.434^**^

^*P< 0.05.^

^**P< 0.01.^

^***P< 0.001.^

By further analysis according to gender, ERα protein expression in neoplastic tissues linked indirectly and significantly with ERβ (r = -0.497; P< 0.0001), PGR (r = -0.158; P< 0.0001), and AR (r = -0.309; P< 0.0001). While ERβ in male malignant tissues correlated positively and moderately with PGR (r = 0.588; P< 0.0001), it showed a weak direct association with AR (r = 0.121; P = 0.002). However, there was no associations between PGR and AR in male malignant tissues. In female tissues, ERα IHC scores in cancerous sites associated inversely with ERβ (r = -0.587; P< 0.0001) and PGR (r = -0.351; P< 0.0001), whilst directly with AR (r = 0.726; P< 0.0001). ERβ protein in female cancer specimens also revealed a direct association with PGR (r = 0.765; P< 0.0001), whereas both inversely linked with AR (r = -0.607 and r = -0.537, respectively; P< 0.0001 for both).

Moreover, ERα and AR in malignant colonic samples showed significant direct associations with tumor size, N stage, numbers of positive regional lymph nodes, and late-stage neoplasms in males, as well as female patients (**Table 3**). In contrast, ERβ and PGR IHC scores in male malignant samples correlated indirectly with N stage, numbers of positive lymph nodes, M stage, lymphovascular invasion and advanced cancer stage, whilst PGR only revealed weak associations with right-sided tumors and poor differentiation (**Table 3**). In female cancerous specimens, both ERβ and PGR exhibited weak to moderate negative correlations with tumor size, N stage, perineural invasion, and late-stage malignancy. Moreover, PGR, but not ERβ, in female malignant specimens correlated indirectly with older age and T stage (**Table 3**).

**Table 3:** Correlations of tumor clinicopathological characteristics with ERα, ERβ, PGR, and AR protein expression in malignant colonic tissues from male (n = 64) and female (n = 56) patients by Pearson’s correlation test.

	Male patients (n = 64)				Female patients (n = 56)			
	*ERα*	*ERβ*	*PGR*	*AR*	*ERα*	*ERβ*	*PGR*	*AR*
**Age**	0.407^**^	-0.031	0.087	0.001	-0.093	0.031	-0.559^**^	-0.023
**Right-sided cancer**	0.143	0.080	-0.292^*^	-0.149	0.109	-0.248	-0.152	0.025
**T stage**	0.031	-0.087	0.051	0.245^*^	0.058	-0.191	-0.349^**^	0.162
**Tumor Size**	0.275^*^	-0.196	-0.218	0.242^*^	0.353^*^	-0.539^**^	-0.429^**^	-0.098
**N Stage**	0.573^***^	-0.694^**^	-0.205	0.360^**^	0.550^**^	-0.201	-0.274^*^	0.654^**^
**Numbers of positive lymph nodes**	0.518^***^	-0.675^**^	-0.195	0.323^**^	0.566^***^	-0.214	-0.180	0.433^**^
**M Stage**	0.186	-0.283^*^	0.024	0.266^*^	.172	-0.273	-0.167	0.338^*^
**Mucinous Carcinoma**	0.039	-0.091	-0.084	0.024	0.096	-0.026	-0.199	0.014
**Poor differentiation**	-0.015	-0.002	0.288^*^	0.015	0.099	0.158	-0.133	0.116
**Lymphovascular invasion**	0.117	-0.304^*^	-0.101	0.043	0.208	-0.126	-0.214	0.376^*^
**Perineural invasion**	-0.084	-0.092	0.044	-0.087	0.222	-0.373^*^	-0.337^*^	0.219
**Late-stage cancer**	0.691^***^	-0.844^***^	-0.175	0.478^**^	0.798^***^	-0.421^**^	-0.465^**^	0.959^***^

^*P< 0.05.^

^**P< 0.01.^

^***P< 0.001.^

### 3.3 *In vitro* effects of sex steroid hormones and their specific receptor blockers

#### 3.3.1 Cytotoxicity and dose-response curves

**Figure 5:**
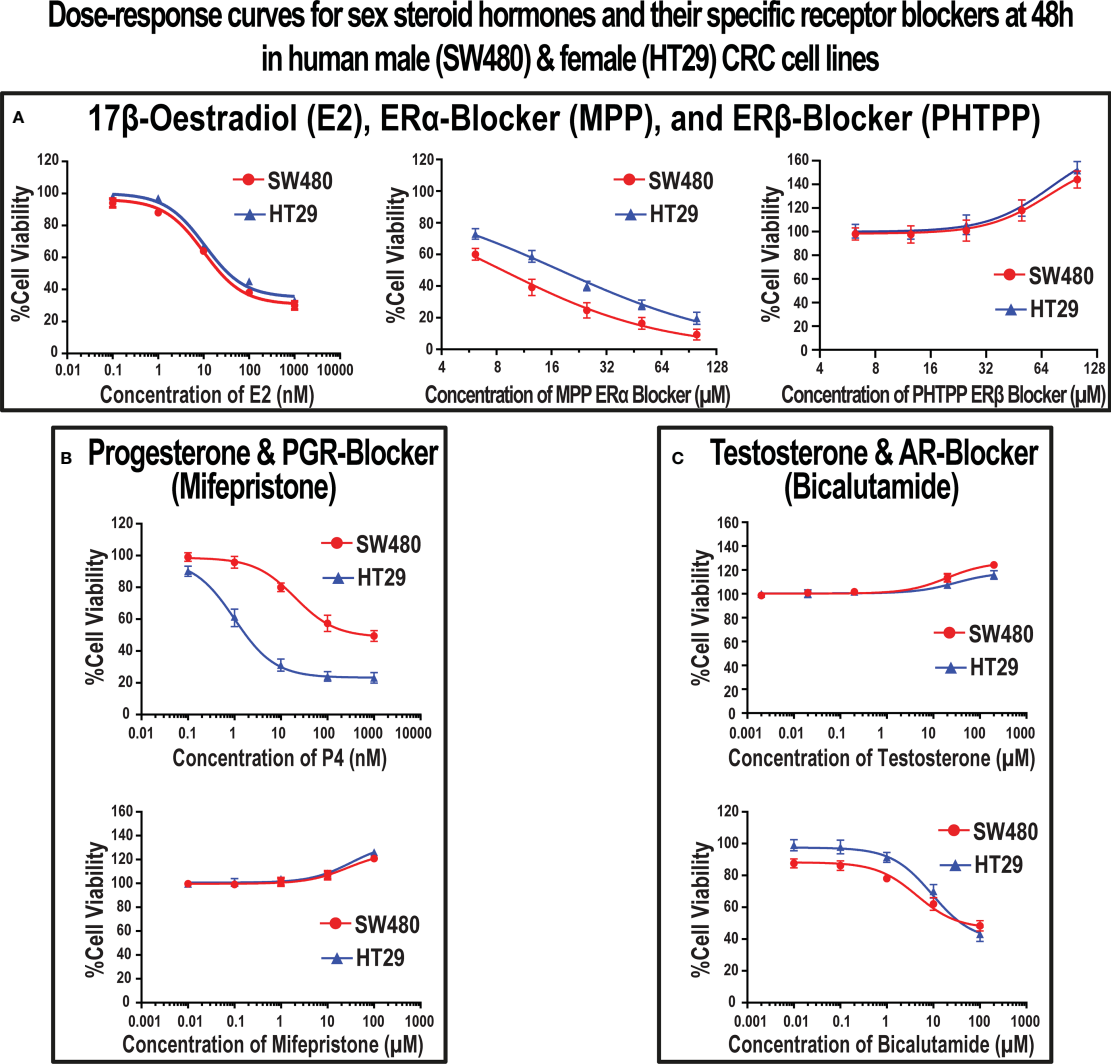
Dose-response curves with the IC50 (mean ± SD) and EC10 (mean ± SD) values of **(A)** 17β-estradiol (E2), ERα-blocker (MPP), and ERβ-blocker (PHTPP), **(B)** progesterone (P4) and PGR-blocker (mifepristone), and **(C)** testosterone and AR-blocker (bicalutamide) at 48h in the SW480 male and HT29 female colon cancer cell lines, as determined using the MTT cell viability assay (Data were analyzed by nonlinear regression to determine dose-response; n = 5 replicates/treatment).

Results of the MTT assay revealed that E2, P4, ERα-blocker (MPP), and AR-blocker (bicalutamide) inhibited proliferation in the SW480 male and HT29 female CRC cell lines (**Figure 5**). Furthermore, the IC50 concentrations were 10 nM for E2 in both cell lines, 20 nM and 1 nM for P4, 8.6 µM and 17.8 µM for MPP, and 4.5 µM and 9.1 µM for bicalutamide in the SW480 and HT29 cells, respectively (**Figure 5**). On the other hand, testosterone, ERβ-blocker (PHTPP), and PGR-blocker (mifepristone) monotherapies promoted cell proliferation in the SW480 and HT29 cell lines. While the EC10 concentrations of PHTPP were 30 µM in both cell lines (**Figure 5A**), they were 26.9 µM and 31.8 µM for mifepristone (**Figure 5B**), and 20 µM and 30 µM for testosterone (**Figure 5C**) in the SW480 and HT29 cells, respectively. Hence, the calculated IC50s of E2, P4, MPP, and bicalutamide, alongside the EC10 of PHTPP, mifepristone, and testosterone, were used to measure their effects on cell cycle and apoptosis in the SW480 and HT29 cells.

#### 3.3.2 Cell cycle progression

**Figure 6:**
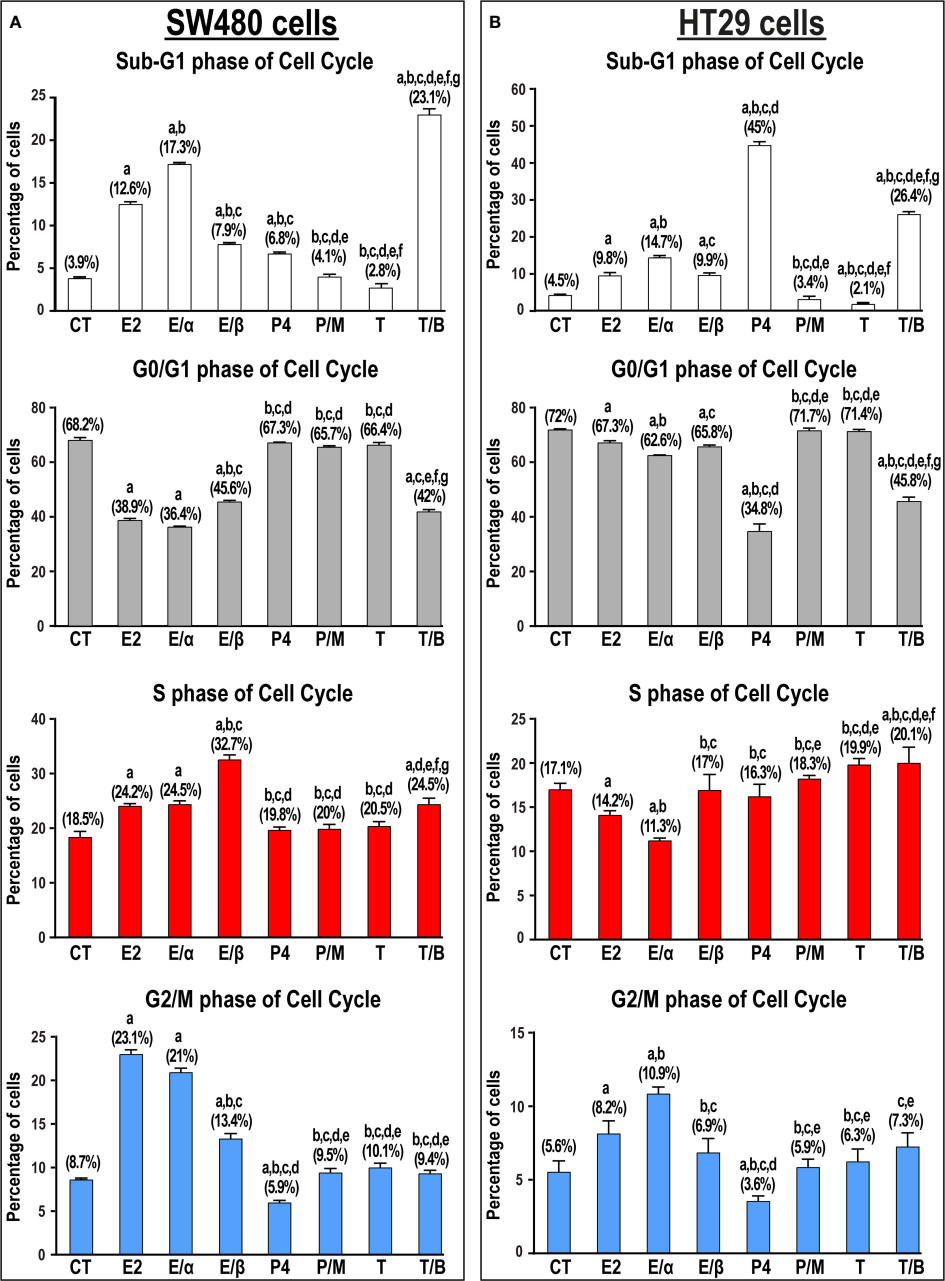
Percentage of cells (mean ± SD) in the different phases of cell cycle in non-treated control cells (CT) and following 17β-estradiol (E2), P4 (P4), and testosterone (T) for 24h alongside dual treatments for 48h with MPP ERα-blocker + E2 (E/α), PHTPP ERβ-blocker + E2 (E/β), mifepristone PGR-blocker + P4 (P/M), and bicalutamide AR-blocker + testosterone (T/B) in the **(A)** SW480 male and **(B)** HT29 female colon cancer cell lines (n = 3 biological replicates/group). Treatment with each receptor blocker was for a total duration of 48h in the co-therapy protocols, whilst E2, P4, or T therapies were incubated for 24h and were initiated in the single and dual treatment groups 24h after adding their corresponding receptor blockers for 24h. (Data were analyzed by one-way ANOVA with Tukey’s HSD *post-hoc* test; a = P< 0.05 compared with CT; b = P< 0.05 compared with E2 group; c = P< 0.05 compared with E/α group, d = P< 0.05 compared with E/β group; e = P< 0.05 compared with P4 group; f = P< 0.05 compared with P/M group, and g = P< 0.05 compared with T group).

Single treatments with E2 and P4 hormones significantly increased the numbers of SW480 (3.2-fold & 1.7-fold, respectively) and HT29 (2.2-fold & 10-fold, respectively) cells in the Sub-G1 phase compared with untreated cells (**Figure 6**). Moreover, ERα-blocker (MPP) markedly increased the percentage of cells relative to non-treated (4.4-fold & 3.3-fold) and E2 monotherapy (1.4-fold & 1.5-fold) in the SW480 and HT29 cell lines, respectively. In contrast, the addition of ERβ-blocker (PHTPP) and PGR-blocker (mifepristone) showed markedly lower numbers of SW480 and HT29 cells in the sub-G1 phase compared with cells treated with E2 and P4 monotherapies (**Figure 6**). Whilst the percentage of SW480 cells in Sub-G1 phase were equal between testosterone monotherapy and untreated cells, the hormone markedly reduced the percentage of HT29 cells in Sub-G1 phase (2.1-fold; **Figure 6**). Nonetheless, the use of AR-blocker (bicalutamide) with testosterone significantly elevated the proportions of SW480 and HT29 cells in Sub-G1 phase relative to control (5.9-fold for both cell lines) and testosterone-only (1.8-fold & 12.6-fold, respectively) groups (**Figure 6**). The scatter and histogram plots, showing the gating strategy used for cell cycle analysis in the SW480 and HT29 cell lines, are represented in **Supplementary Figures 5**, **6**, respectively.

E2-alone or combined with ERα-blocker also induced arrest at the S and G2/M phases of cell cycle in the SW480 cells, whilst only promoting G2/M-arrest in the HT29 cells (**Figure 6**). Moreover, the combination of E2 with its ERβ-blocker was associated with S-phase arrest in the SW480, but not HT29, cells. In contrast, P4, with and without mifepristone, displayed negligible effects on the numbers of SW480 and HT29 cells in the different phases of cell cycle (**Figure 6**). On the other hand, testosterone and bicalutamide co-therapy induced marked increases in the numbers of SW480 and HT29 cells in the S-phase of cell cycle, with concomitant declines in the percentage of cells in the G0/G1 and G2/M phases (**Figure 6**).

#### 3.3.3 Cell apoptosis

**Figure 7:**
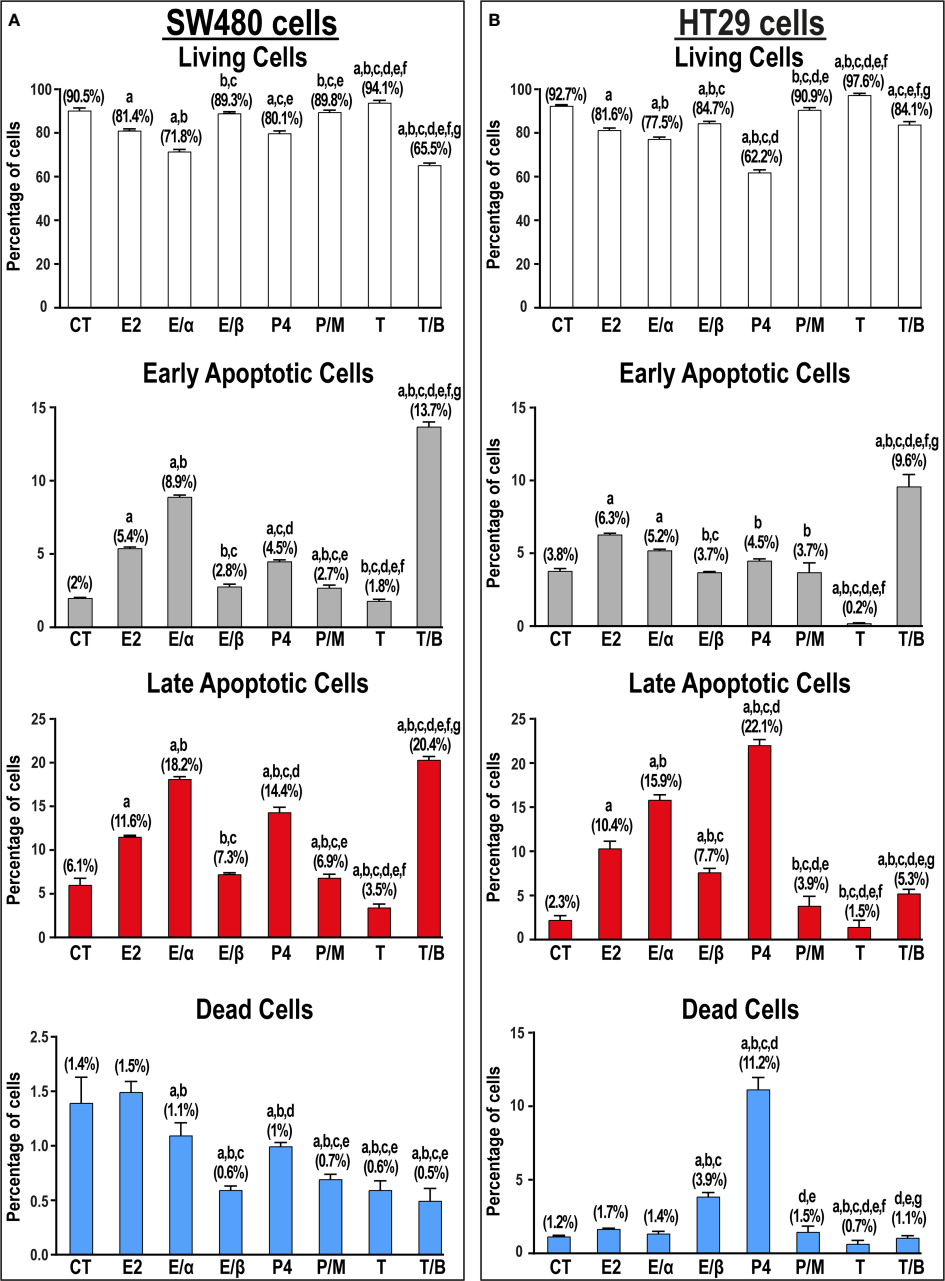
Percentage (mean ± SD) of living, early and late apoptotic alongside dead cells in non-treated control cells (CT) and following 17β-estradiol (E2), P4 (P4), and testosterone (T) for 24h alongside dual treatments for 48h with MPP ERα-blocker + E2 (E/α), PHTPP ERβ-blocker + E2 (E/β), mifepristone PGR-blocker + P4 (P/M), and bicalutamide AR-blocker + testosterone (T/B) in the **(A)** SW480 male and **(B)** HT29 female colon cancer cell lines (n = 3 biological replicates/group). Treatment with each receptor blocker was for a total duration of 48h in the co-therapy protocols, whilst E2, P4, or T therapies were incubated for 24h and were initiated in the single and dual treatment groups 24h after adding their corresponding receptor blockers for 24h. (Data were analyzed by one-way ANOVA with Tukey’s HSD *post-hoc* test; a = P< 0.05 compared with CT; b = P< 0.05 compared with E2 group; c = P< 0.05 compared with E/α group, d = P< 0.05 compared with E/β group; e = P< 0.05 compared with P4 group; f = P< 0.05 compared with P/M group, and g = P< 0.05 compared with T group).

E2 monotherapy significantly reduced the numbers of viable SW480 and HT29 cells that was depicted by marked increases in the percentage of early (2.7-fold & 1.6-fold, respectively) and late (1.9-fold & 4.5-fold, respectively) apoptotic cells relative to untread cells (**Figure 7**). The addition of ERα-blocker significantly boosted, whilst ERβ-blocker inhibited, the pro-apoptotic effects of E2 therapy in both cell lines (**Figure 7**). Similarly, P4 single treatment markedly reduced cell viability by increasing the percentage of early (2.3-fold & 1.2-fold) and late (2.4-fold & 9.6-fold) apoptotic SW480 and HT29 cells, respectively, and the effects were inhibited by the PGR-blocker, mifepristone (**Figure 7**). In contrast, testosterone monotherapy significantly increased the numbers of viable SW480 and HT29 cells, whilst its combination with bicalutamide showed marked elevations in the percentage of early and late apoptotic SW480 (6.9-fold & 3.3-fold, respectively) and HT29 (2.5-fold & 2.3-fold) cells (**Figure 7**).

## 4 Discussion

Herein, we measured ERα, ERβ, PGR, and AR protein expression in archived paired malignant and non-malignant colonic tissues, and the results were analyzed based on gender, age, and tumor sidedness. We also measured the effects of E2, P4, and testosterone, with and without their corresponding specific nuclear receptor blockers, on cell cycle and apoptosis in the SW480 male and HT29 female CRC cell lines. Our results revealed gender-dependent protein expression of the targeted sex steroid receptors in non-cancerous colonic tissues. Moreover, ERα and AR proteins increased, whereas ERβ and PGR diminished markedly in neoplastic relative to non-neoplastic colonic tissues, and the dysregulations were maximal in late-stage cancers in both genders. ERα and AR proteins also correlated directly, whilst ERβ and PGR negatively, with tumors’ histopathological features. Concomitantly, E2 monotherapy induced cell cycle arrest and promoted apoptosis in the SW480 male and HT29 female CRC cell lines, and pre-treating with ERα-blocker enhanced, whereas ERβ-blocker inhibited the anticancer actions. Similarly, P4 induced apoptosis in both cell lines, whilst adding the PGR-blocker impeded the effects. In contrast, testosterone promoted survival in both cell lines, and the effects were hindered by the specific AR-blocker, bicalutamide. However, the best apoptotic actions in the SW480 male cell line were detected after blocking AR, whilst P4 showed the highest pro-apoptotic effects in the HT29 female cell line.

The importance of sex steroid hormones in colon cancer has gained greater attention, since the rates of CRC in premenopausal, as well as post-menopausal women using HRT, were markedly lower than nonuser post-menopausal women and age-matched men (6, 8, 9, 44). The risk of CRC also increased significantly in women after oophorectomy (45, 46), and 17β-estradiol treatment *in vivo* and *in vitro* inhibited CRC progression *via* ERβ-mediated actions (33–36), whilst promoted cancer progression through ERα (47–49). Others have likewise reported lower CRC incidence in menopausal women using P4 (50, 51), and the hormone also triggered cell cycle arrest and apoptosis in several human CRC cell lines, including SW480 and HT29 cells (30, 36, 52). Furthermore, ERβ expression declined significantly in malignant relative to non-malignant colonic tissues and coincided with drastically elevated ERα (48, 53–55), and the expression linked with lower survival and higher recurrence rates, advanced stages, and distant metastasis (23–25, 27–29). Although many reports also disclosed marked declines in PGR in cancerous tissues, their results related to its prognostic value in CRC are inconclusive (23, 30–32). In contrast, male rats were more susceptible for developing CRC than females (37, 38), and orchiectomy reduced, whereas testosterone replacement therapy sustained, the numbers of tumors in male animals (38). Similar findings were also reported after chemically inducing CRC in male mice (39), as well as testosterone caused dose-dependent increases in the viability of HT29 cells (40). Moreover, AR increased in malignant clinical samples and correlated directly with tumor size, poor differentiation, lymph node positivity, advanced stage, and poor prognosis (26). However, the authors did not compare the expression between both genders. On the other hand, increased AR in female malignant tissues has been suggested to promote CRC aggressiveness by increasing classes III and V of β-tubulin protein, which are commonly activated in male patients and could underly the observed higher mortality rate in men (56).

Collectively, Our findings correlate with earlier studies reporting gender-dependent expression of ERs (14–16), PGR (17–19), and AR (20–22) in normal colon, which supports the notion that sex steroid hormones contribution to colon biology could, at least in part, be gender-specific. Moreover, our data and prior studies advocate that ERβ (33–36) and PGR (30, 36, 52) mediate tumor suppressive actions, whereas overexpressed ERα (23–25) and AR (37–40) could incite oncogenicity in colon. The present findings also provide additional support for the potential prognostic values of sex steroid receptors in CRC (23–30, 32). Although the current results also reinforce the notion that hormonal therapy could represent an alternative therapy for CRC (36, 38, 39, 50, 51, 57), treatment regimens should be tailored based on gender alongside the expression of sex steroid receptors in malignant tissues to achieve the highest efficacy. In more detail, we suggest that the use of ovarian sex steroid hormones (33–36, 50, 51), as well as blocking androgen receptor (38–40), could inhibit CRC progression by modulating the regulatory molecules of cell cycle and apoptosis. Nonetheless, further studies using the different sex steroid hormones and/or their specific receptor blockers, with and without 5-Fluororacil, are mandatory to measure their therapeutic efficiencies against CRC. Additionally, more studies are needed to elucidate the roles of sex steroid hormones in colon neoplasia by measuring their effects on the expression of oncogenic and tumor suppressive molecules.

Tumor sidedness is another important factor in CRC, and RSCs are linked with older age, poor differentiation, mucinous carcinoma, and worse outcomes (3–5). The oncogenic pathways and molecular features also vary substantially between proximal and distal colonic neoplasms (3–5). Despite this, little is known about the expression of sex steroid receptors in right and left-sided cancers. Herein, there was an overall significant increase in the expression of ERα, whilst PGR declined, in LSCs compared to RSCs. Furthermore, the highest ERα alongside the lowest PGR protein expression were observed in women aged ≥ 60 years and diagnosed with late-stage LSCs. On the other hand, ERβ and AR proteins were in general equal between both anatomical sides. By disseminating the data according to gender, however, late-stage LSCs obtained from females ≥ 60 years of age showed the weakest and strongest protein expression of ERβ and AR, respectively. In contrast, right and left-sided male malignant tissues exhibited equivalent expression of ERβ, as well as AR, in the different clinical stages of CRC.

Taken together, our findings suggest that ERα and PGR protein expression in CRC could be reliant on tumor sidedness alongside gender, age, and clinical stage. Paradoxically, loss of ERβ and AR overexpression in malignant tissues appear to be gender-specific, since in male patients the deregulations were constant between RSCs and LSCs at the different stages, whilst in post-menopausal female patients the alterations were more pronounced in distal colon cancers, especially during the late stages. Hence, we speculate that the rates of CRC are lower in premenopausal women (8, 9, 44) due to higher ERβ (14, 33, 35) and PGR (30, 52) alongside lower ERα (47–49) and AR (56, 58) expression in colonic tissues, whereas their pathological alterations following menopause might trigger CRC. Moreover, future studies should consider gender, age, clinical stage, and tumor sidedness to precisely explore the roles of the targeted sex steroid receptors and/or measure their prognostic values in CRC.

This study has several drawbacks. Firstly, we only measured the protein expression of the targeted receptors, and future studies should also measure their gene expression. Moreover, the patients included had their surgical innervations between January 2019 and December 2021 and, therefore, prognostic data (e.g., 5-year survival rate, disease-free survival rate, etc.), as well as data related to using hormone replacement therapy were not available to correlate them with the expression profiles of the targeted receptors. Hence, additional studies are still needed to measure the prognostic values of sex steroid receptors according to gender, tumor sites, and clinical stages. Furthermore, we only measured the expression of the targeted receptors by IHC, and future prospective studies that include fresh tissues are needed to validate the protein expression by additional techniques (e.g., Western blot). More *in vitro* studies are also required to investigate the molecular pathways underlying the actions of sex steroid hormones and their receptors in CRC (e.g., cell cycle regulatory molecules, apoptosis regulatory molecules, etc.). Moreover, future studies should also measure the genes and proteins of the G-protein coupled membrane (e.g., mER, mPGR, and mAR) and the nuclear receptors, since the membranous receptors were shown to mediate anti-tumorigenic actions in CRC (59, 60).

In conclusion, non-malignant tissues from women ≤ 50 years of age showed markedly lower ERα and AR alongside stronger ERβ and PGR proteins than men and women aged ≥ 60 years, which could explain the commonly reported lower CRC incidence in premenopausal women. In malignant tissues, the proteins of ERα and AR increased significantly and concurred with decreases in ERβ and PGR, and the tumor clinical characteristics correlated positively with ERα and AR, whilst negatively with ERβ and PGR, supporting the contributions of receptors to colon carcinogenesis. However, the expression profiles of the sex steroid receptors in cancerous tissues varied between genders, clinical stages, and tumor sidedness. Moreover, E2 and P4 monotherapies induced apoptosis, whilst testosterone caused proliferation in the SW480 male and HT29 female CRC cell lines, and the effects were reversed by pre-treating the cells with the specific blockers of ERβ, PGR, and AR receptors, respectively. Collectively, this study advocates the promising prognostic value of the targeted sex steroid receptors, as well as the potential benefits of hormonal therapy in CRC. However, future studies should measure the expression of membranous and nuclear receptors of sex steroid hormones in malignant colonic tissues and the results should be disseminated according to gender, age, clinical stage, and tumor sidedness to accurately determine their prognostic values in CRC. More *in vivo* and *in vitro* studies are also still needed to measure the anti-cancer effects of sex steroid hormones and their receptor blockers, with and without chemotherapy, to precisely tailor hormonal therapeutic regimens against CRC based on gender and the expression of sex steroid receptors.

## Data availability statement

The original contributions presented in the study are included in the article/**Supplementary Material**. Further inquiries can be directed to the corresponding author.

## Ethics statement

The studies involving human participants were reviewed and approved by The Institutional Review Board of King Abdullah Medical City in Makkah, Saudi Arabia (KAMC; #19-498). Written informed consent for participation was not required for this study in accordance with the national legislation and the institutional requirements.

## Author contributions

Conceptualization: BR, AkA, and SI. Methodology: AkA, SI, AhA, MofA, HA, GB, OA and IM. Investigation: AkA. RA, FM and BR. Visualization: BR, SA, MonA, BB and MaA. Validation: SA, MonA, BB and MaA. Formal analysis: BR, AkA, RA, and FM. Data curation: BR, AkA. and SI. Supervision: BR, AkA, RA and FM. Funding acquisition: BR, AkA, RA and FM. Resources: BR and AkA. Project administration: BR RA and FM. Writing—original draft: BR AhA, MofA and HA. Writing—review and editing: AkA. All authors contributed to the article and approved the submitted version.

## Supplementary material

Supplementary Figure 1
**(A)** Immunohistochemical localization of ERα in non-malignant colonic tissues collected from patients diagnosed with early-stage (I/II) and late-stage (III/IV) colorectal cancer (20× objective; Scale bar = 15 μm) alongside **(B)** their IHC arbitrary scores are shown as boxplots according to gender, age, tumor sides, and cancer stages. (a = P< 0.05 compared with males ≤ 50 years with right sided early-stage cancer; b = P< 0.05 compared with males ≥ 60 years with right sided early-stage cancer; c = P< 0.05 compared with females ≤ 50 years with right sided early-stage cancer; d = P< 0.05 compared with females ≥ 60 years with right sided early-stage cancer; e = P< 0.05 compared with males ≤ 50 years with right sided late-stage cancer; f = P< 0.05 compared with males ≥ 60 years with right sided late-stage cancer; g = P< 0.05 compared with females ≤ 50 years with right sided late-stage cancer; h = P< 0.05 compared with females ≥ 60 years with right sided late-stage cancer; i = P< 0.05 compared with males ≤ 50 years with left sided early-stage cancer; j = P< 0.05 compared with males ≥ 60 years with left sided early-stage cancer; k = P< 0.05 compared with females ≤ 50 years with left sided early-stage cancer; l = P< 0.05 compared with females ≥ 60 years with left sided early-stage cancer; m = P< 0.05 compared with males ≤ 50 years with left sided late-stage cancer; n = P< 0.05 compared with males ≥ 60 years with left sided late-stage cancer and o = P< 0.05 compared with females ≤ 50 years with left sided late-stage cancer).

Supplementary Figure 2
**(A)** Immunohistochemical localization of ERβ in non-malignant colonic tissues collected from patients diagnosed with early-stage (I/II) and late-stage (III/IV) colorectal cancer (20× objective; Scale bar = 15 μm) alongside **(B)** their IHC arbitrary scores are shown as boxplots according to gender, age, tumor sides, and cancer stages. (a = P< 0.05 compared with males ≤ 50 years with right sided early-stage cancer; b = P< 0.05 compared with males ≥ 60 years with right sided early-stage cancer; c = P< 0.05 compared with females ≤ 50 years with right sided early-stage cancer; d = P< 0.05 compared with females ≥ 60 years with right sided early-stage cancer; e = P< 0.05 compared with males ≤ 50 years with right sided late-stage cancer; f = P< 0.05 compared with males ≥ 60 years with right sided late-stage cancer; g = P< 0.05 compared with females ≤ 50 years with right sided late-stage cancer; h = P< 0.05 compared with females ≥ 60 years with right sided late-stage cancer; i = P< 0.05 compared with males ≤ 50 years with left sided early-stage cancer; j = P< 0.05 compared with males ≥ 60 years with left sided early-stage cancer; k = P< 0.05 compared with females ≤ 50 years with left sided early-stage cancer; l = P< 0.05 compared with females ≥ 60 years with left sided early-stage cancer; m = P< 0.05 compared with males ≤ 50 years with left sided late-stage cancer; n = P< 0.05 compared with males ≥ 60 years with left sided late-stage cancer and o = P< 0.05 compared with females ≤ 50 years with left sided late-stage cancer).

Supplementary Figure 3
**(A)** Immunohistochemical localization of PGR in non-malignant colonic tissues collected from patients diagnosed with early-stage (I/II) and late-stage (III/IV) colorectal cancer (20× objective; Scale bar = 15 μm) alongside **(B)** their IHC arbitrary scores are shown as boxplots according to gender, age, tumor sides, and cancer stages. (a = P< 0.05 compared with males ≤ 50 years with right sided early-stage cancer; b = P< 0.05 compared with males ≥ 60 years with right sided early-stage cancer; c = P< 0.05 compared with females ≤ 50 years with right sided early-stage cancer; d = P< 0.05 compared with females ≥ 60 years with right sided early-stage cancer; e = P< 0.05 compared with males ≤ 50 years with right sided late-stage cancer; f = P< 0.05 compared with males ≥ 60 years with right sided late-stage cancer; g = P< 0.05 compared with females ≤ 50 years with right sided late-stage cancer; h = P< 0.05 compared with females ≥ 60 years with right sided late-stage cancer; i = P< 0.05 compared with males ≤ 50 years with left sided early-stage cancer; j = P< 0.05 compared with males ≥ 60 years with left sided early-stage cancer; k = P< 0.05 compared with females ≤ 50 years with left sided early-stage cancer; l = P< 0.05 compared with females ≥ 60 years with left sided early-stage cancer; m = P< 0.05 compared with males ≤ 50 years with left sided late-stage cancer; n = P< 0.05 compared with males ≥ 60 years with left sided late-stage cancer and o = P< 0.05 compared with females ≤ 50 years with left sided late-stage cancer).

Supplementary Figure 4
**(A)** Immunohistochemical localization of AR in non-malignant colonic tissues collected from patients diagnosed with early-stage (I/II) and late-stage (III/IV) colorectal cancer (20× objective; Scale bar = 15 μm) alongside **(B)** their IHC arbitrary scores are shown as boxplots according to gender, age, tumor sides, and cancer stages. (a = P< 0.05 compared with males ≤ 50 years with right sided early-stage cancer; b = P< 0.05 compared with males ≥ 60 years with right sided early-stage cancer; c = P< 0.05 compared with females ≤ 50 years with right sided early-stage cancer; d = P< 0.05 compared with females ≥ 60 years with right sided early-stage cancer; e = P< 0.05 compared with males ≤ 50 years with right sided late-stage cancer; f = P< 0.05 compared with males ≥ 60 years with right sided late-stage cancer; g = P< 0.05 compared with females ≤ 50 years with right sided late-stage cancer; h = P< 0.05 compared with females ≥ 60 years with right sided late-stage cancer; i = P< 0.05 compared with males ≤ 50 years with left sided early-stage cancer; j = P< 0.05 compared with males ≥ 60 years with left sided early-stage cancer; k = P< 0.05 compared with females ≤ 50 years with left sided early-stage cancer; l = P< 0.05 compared with females ≥ 60 years with left sided early-stage cancer; m = P< 0.05 compared with males ≤ 50 years with left sided late-stage cancer; n = P< 0.05 compared with males ≥ 60 years with left sided late-stage cancer and o = P< 0.05 compared with females ≤ 50 years with left sided late-stage cancer).

Supplementary Figure 5 Cell cycle analysis data for SW480 cells with the gating strategy used for each treatment group. The proportion of each phase of the cell cycle was determined for 20,000 single cell events using the NovoExpress cell cycle algorithm (right panel; histogram), and first gated on the SW480 cell population using forward scatter (FSC) vs side scatter (SSC) scatter plots (left panels), and then using DNA content height (H) *vs.* Area (A) scatter plots (middle panels) to calculate single cell events (pulse processing). The plots shown are representative of one of three similar experiments, and the percentage of each cell cycle phase is shown (mean ± SD; n = 3).

Supplementary Figure 6 Cell cycle analysis data for HT29 cells with the gating strategy used for each treatment group. The proportion of each phase of the cell cycle was determined for 20,000 single cell events using the NovoExpress cell cycle algorithm (right panel; histogram), and first gated on the HT29 cell population using forward scatter (FSC) vs side scatter (SSC) scatter plots (left panels), and then using DNA content height (H) *vs.* Area (A) scatter plots (middle panels) to calculate single cell events (pulse processing). The plots shown are representative of one of three similar experiments, and the percentage of each cell cycle phase is shown (mean ± SD; n = 3).
