# Supplementary figures and images for: Profiling estrogen, progesterone, and androgen receptors in colorectal cancer in relation to gender, menopausal status, clinical stage, and tumour sidedness
Source: Front Endocrinol (Lausanne). 2023 May 3;14:1187259. doi: 10.3389/fendo.2023.1187259 (PMC10190606; doi:10.3389/fendo.2023.1187259)

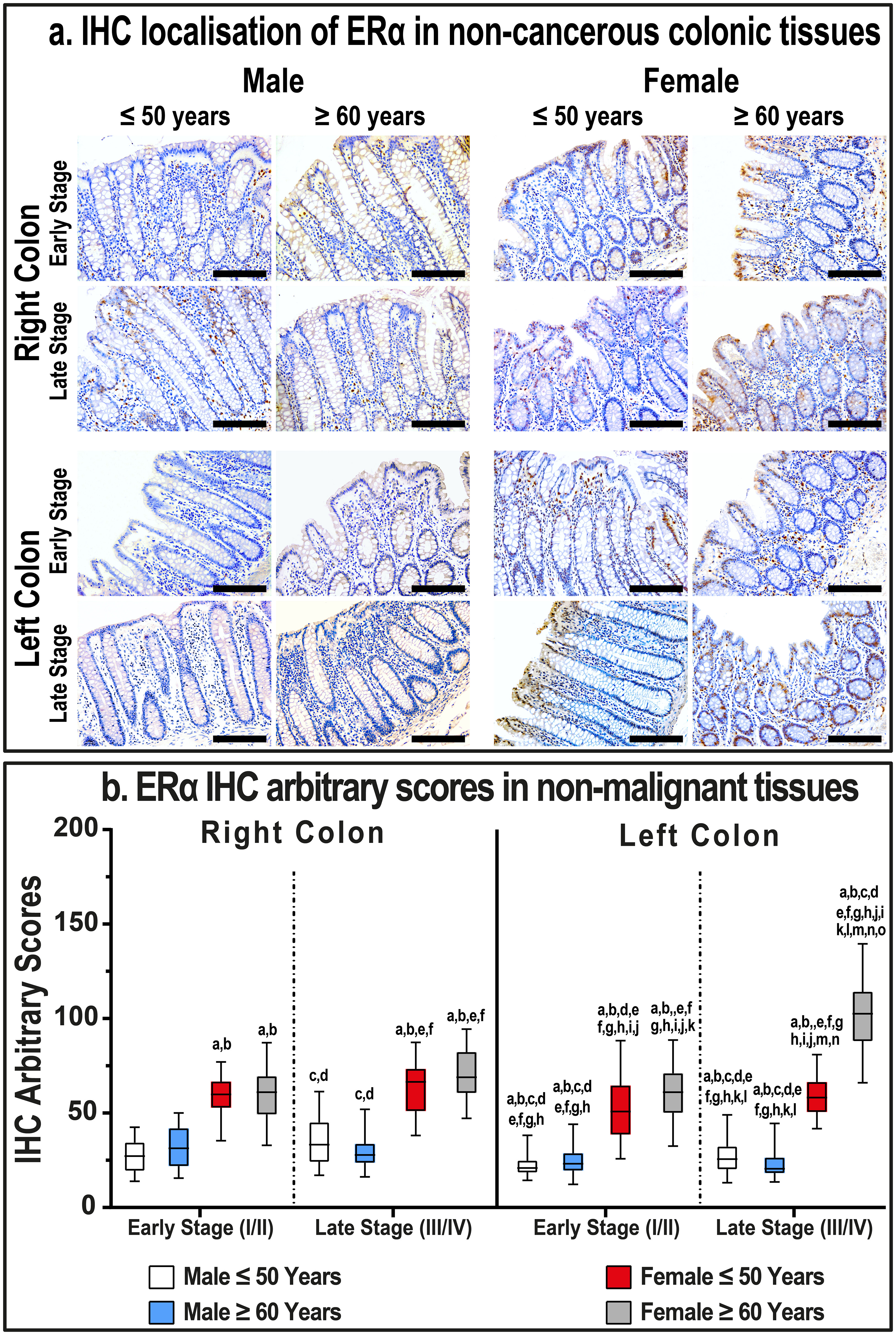

Supplement: Supplementary Figure 1 — (A) Immunohistochemical localization of ERα in non-malignant colonic tissues collected from patients diagnosed with early-stage (I/II) and late-stage (III/IV) colorectal cancer (20× objective; Scale bar = 15 μm) alongside (B) their IHC arbitrary scores are shown as boxplots according to gender, age, tumor sides, and cancer stages. (a = P< 0.05 compared with males ≤ 50 years with right sided early-stage cancer; b = P< 0.05 compared with males ≥ 60 years with right sided early-stage cancer; c = P< 0.05 compared with females ≤ 50 years with right sided early-stage cancer; d = P< 0.05 compared with females ≥ 60 years with right sided early-stage cancer; e = P< 0.05 compared with males ≤ 50 years with right sided late-stage cancer; f = P< 0.05 compared with males ≥ 60 years with right sided late-stage cancer; g = P< 0.05 compared with females ≤ 50 years with right sided late-stage cancer; h = P< 0.05 compared with females ≥ 60 years with right sided late-stage cancer; i = P< 0.05 compared with males ≤ 50 years with left sided early-stage cancer; j = P< 0.05 compared with males ≥ 60 years with left sided early-stage cancer; k = P< 0.05 compared with females ≤ 50 years with left sided early-stage cancer; l = P< 0.05 compared with females ≥ 60 years with left sided early-stage cancer; m = P< 0.05 compared with males ≤ 50 years with left sided late-stage cancer; n = P< 0.05 compared with males ≥ 60 years with left sided late-stage cancer and o = P< 0.05 compared with females ≤ 50 years with left sided late-stage cancer). [file Image_1.jpeg]

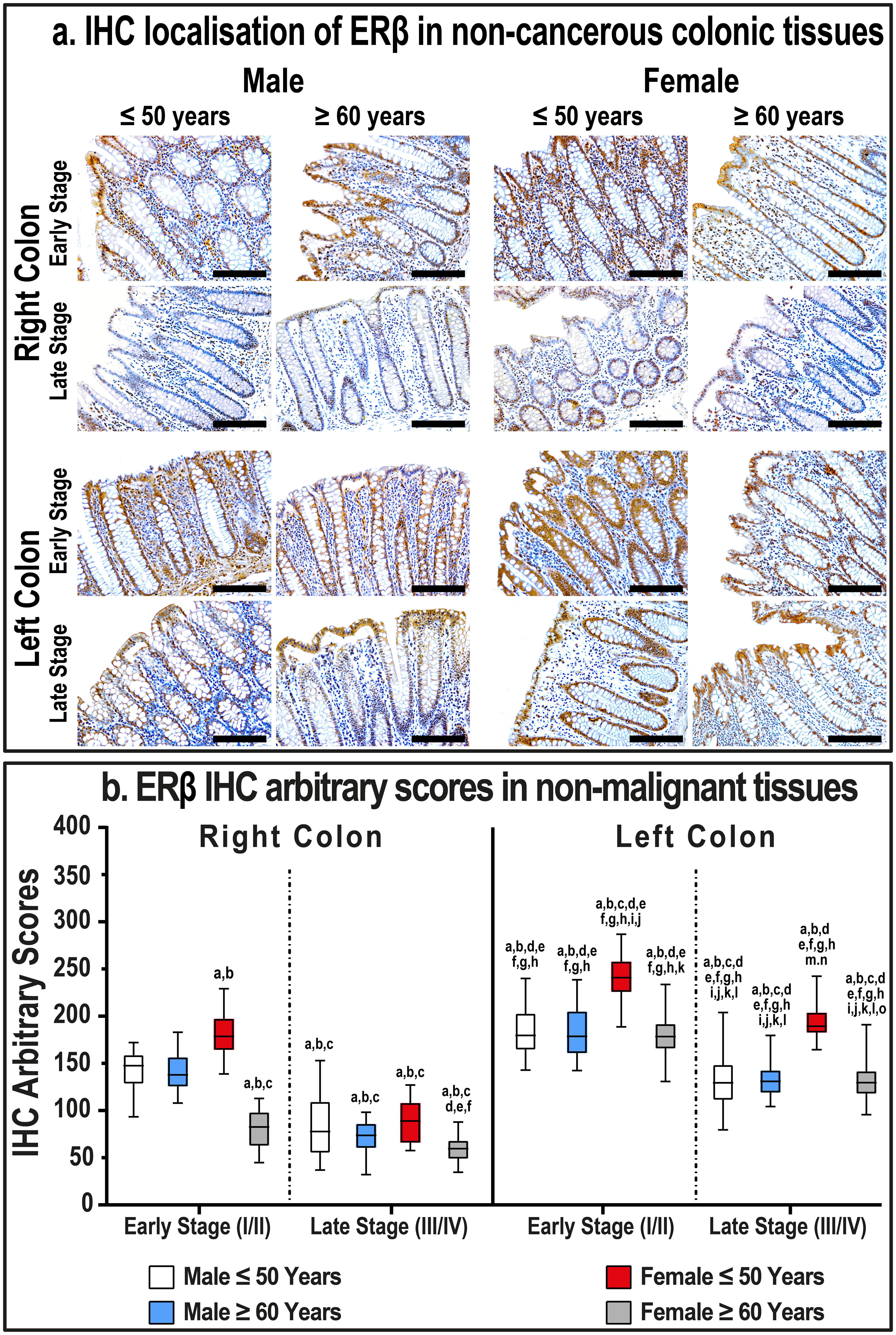

Supplement: Supplementary Figure 2 — (A) Immunohistochemical localization of ERβ in non-malignant colonic tissues collected from patients diagnosed with early-stage (I/II) and late-stage (III/IV) colorectal cancer (20× objective; Scale bar = 15 μm) alongside (B) their IHC arbitrary scores are shown as boxplots according to gender, age, tumor sides, and cancer stages. (a = P< 0.05 compared with males ≤ 50 years with right sided early-stage cancer; b = P< 0.05 compared with males ≥ 60 years with right sided early-stage cancer; c = P< 0.05 compared with females ≤ 50 years with right sided early-stage cancer; d = P< 0.05 compared with females ≥ 60 years with right sided early-stage cancer; e = P< 0.05 compared with males ≤ 50 years with right sided late-stage cancer; f = P< 0.05 compared with males ≥ 60 years with right sided late-stage cancer; g = P< 0.05 compared with females ≤ 50 years with right sided late-stage cancer; h = P< 0.05 compared with females ≥ 60 years with right sided late-stage cancer; i = P< 0.05 compared with males ≤ 50 years with left sided early-stage cancer; j = P< 0.05 compared with males ≥ 60 years with left sided early-stage cancer; k = P< 0.05 compared with females ≤ 50 years with left sided early-stage cancer; l = P< 0.05 compared with females ≥ 60 years with left sided early-stage cancer; m = P< 0.05 compared with males ≤ 50 years with left sided late-stage cancer; n = P< 0.05 compared with males ≥ 60 years with left sided late-stage cancer and o = P< 0.05 compared with females ≤ 50 years with left sided late-stage cancer). [file Image_2.jpeg]

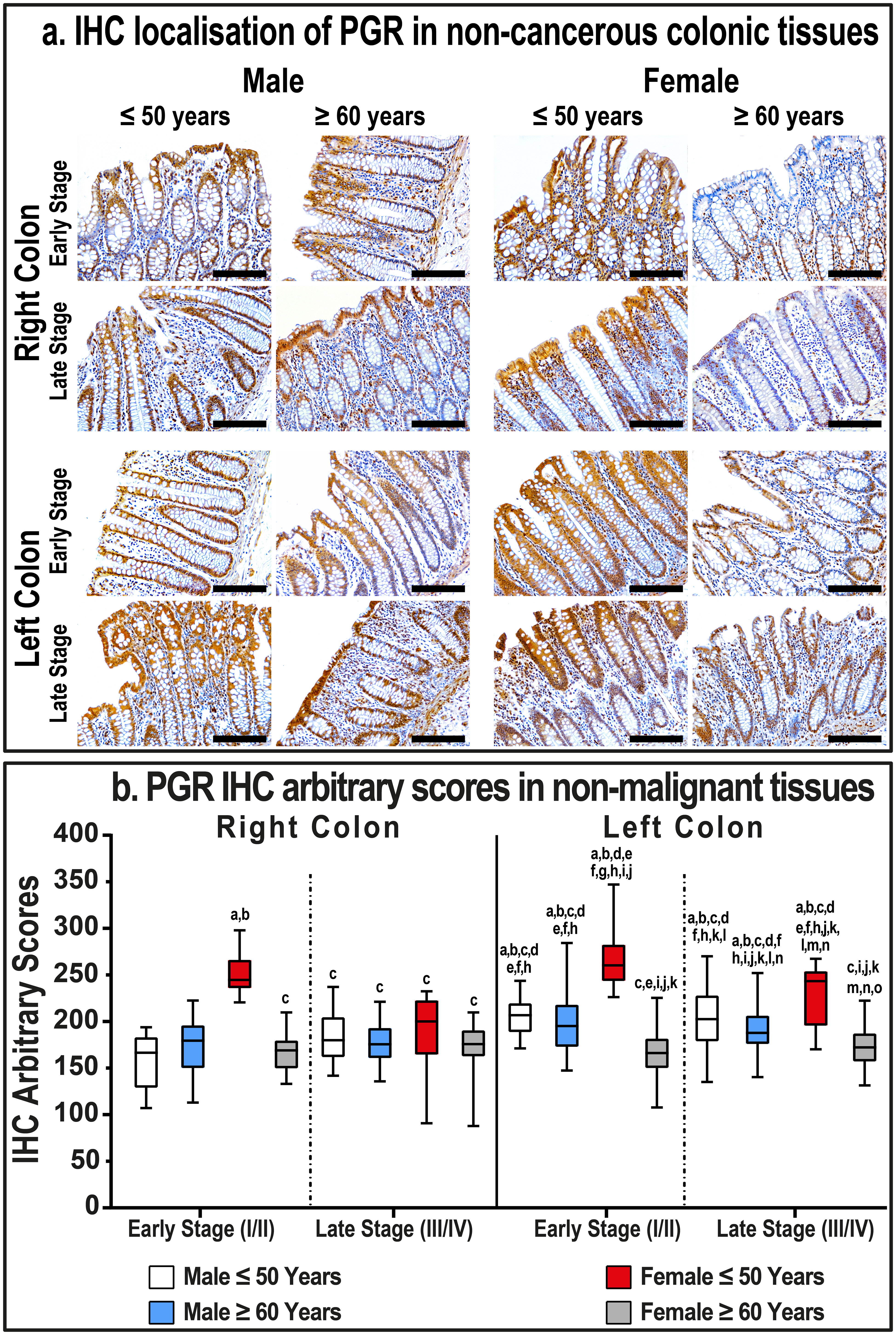

Supplement: Supplementary Figure 3 — (A) Immunohistochemical localization of PGR in non-malignant colonic tissues collected from patients diagnosed with early-stage (I/II) and late-stage (III/IV) colorectal cancer (20× objective; Scale bar = 15 μm) alongside (B) their IHC arbitrary scores are shown as boxplots according to gender, age, tumor sides, and cancer stages. (a = P< 0.05 compared with males ≤ 50 years with right sided early-stage cancer; b = P< 0.05 compared with males ≥ 60 years with right sided early-stage cancer; c = P< 0.05 compared with females ≤ 50 years with right sided early-stage cancer; d = P< 0.05 compared with females ≥ 60 years with right sided early-stage cancer; e = P< 0.05 compared with males ≤ 50 years with right sided late-stage cancer; f = P< 0.05 compared with males ≥ 60 years with right sided late-stage cancer; g = P< 0.05 compared with females ≤ 50 years with right sided late-stage cancer; h = P< 0.05 compared with females ≥ 60 years with right sided late-stage cancer; i = P< 0.05 compared with males ≤ 50 years with left sided early-stage cancer; j = P< 0.05 compared with males ≥ 60 years with left sided early-stage cancer; k = P< 0.05 compared with females ≤ 50 years with left sided early-stage cancer; l = P< 0.05 compared with females ≥ 60 years with left sided early-stage cancer; m = P< 0.05 compared with males ≤ 50 years with left sided late-stage cancer; n = P< 0.05 compared with males ≥ 60 years with left sided late-stage cancer and o = P< 0.05 compared with females ≤ 50 years with left sided late-stage cancer). [file Image_3.jpeg]

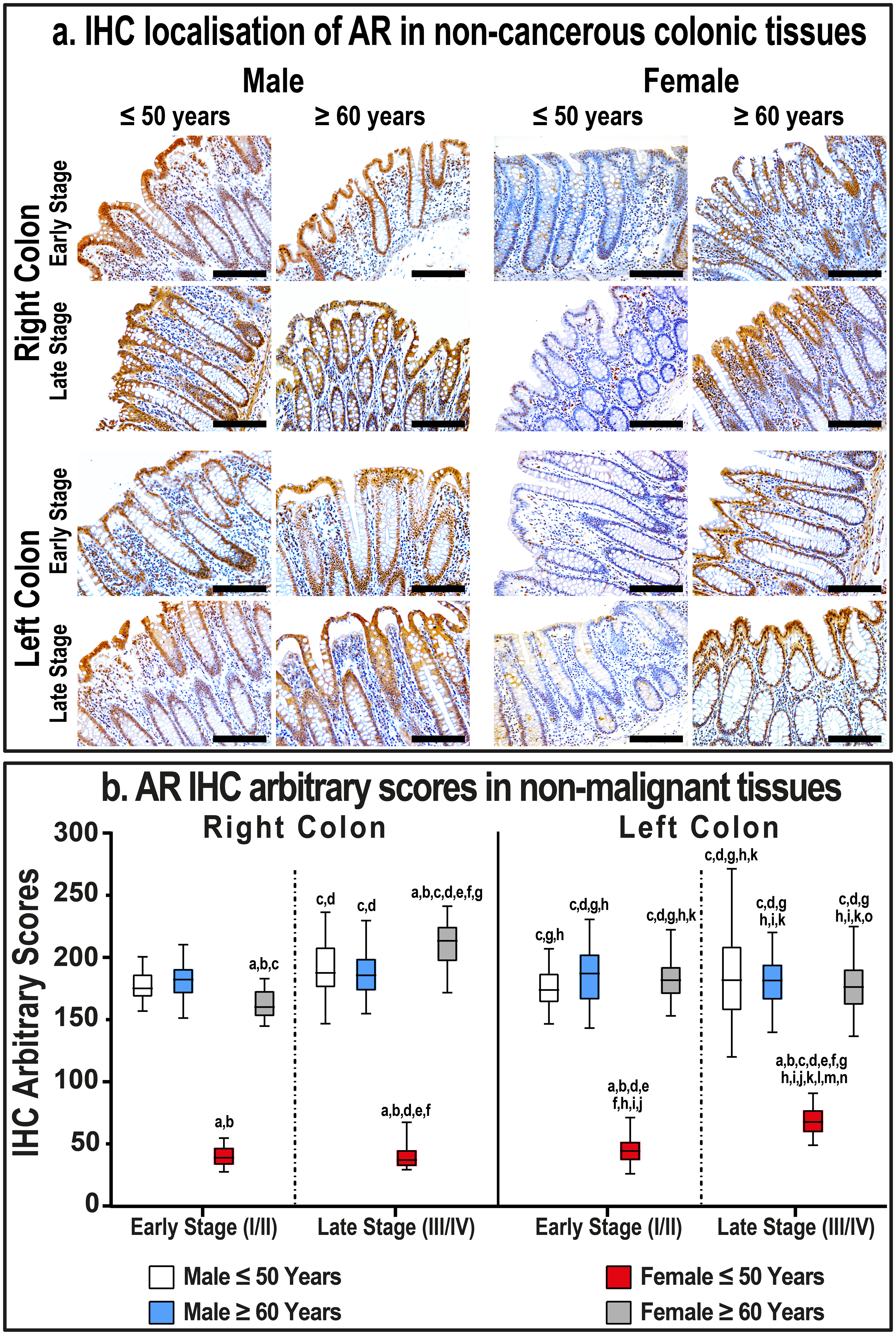

Supplement: Supplementary Figure 4 — (A) Immunohistochemical localization of AR in non-malignant colonic tissues collected from patients diagnosed with early-stage (I/II) and late-stage (III/IV) colorectal cancer (20× objective; Scale bar = 15 μm) alongside (B) their IHC arbitrary scores are shown as boxplots according to gender, age, tumor sides, and cancer stages. (a = P< 0.05 compared with males ≤ 50 years with right sided early-stage cancer; b = P< 0.05 compared with males ≥ 60 years with right sided early-stage cancer; c = P< 0.05 compared with females ≤ 50 years with right sided early-stage cancer; d = P< 0.05 compared with females ≥ 60 years with right sided early-stage cancer; e = P< 0.05 compared with males ≤ 50 years with right sided late-stage cancer; f = P< 0.05 compared with males ≥ 60 years with right sided late-stage cancer; g = P< 0.05 compared with females ≤ 50 years with right sided late-stage cancer; h = P< 0.05 compared with females ≥ 60 years with right sided late-stage cancer; i = P< 0.05 compared with males ≤ 50 years with left sided early-stage cancer; j = P< 0.05 compared with males ≥ 60 years with left sided early-stage cancer; k = P< 0.05 compared with females ≤ 50 years with left sided early-stage cancer; l = P< 0.05 compared with females ≥ 60 years with left sided early-stage cancer; m = P< 0.05 compared with males ≤ 50 years with left sided late-stage cancer; n = P< 0.05 compared with males ≥ 60 years with left sided late-stage cancer and o = P< 0.05 compared with females ≤ 50 years with left sided late-stage cancer). [file Image_4.jpeg]

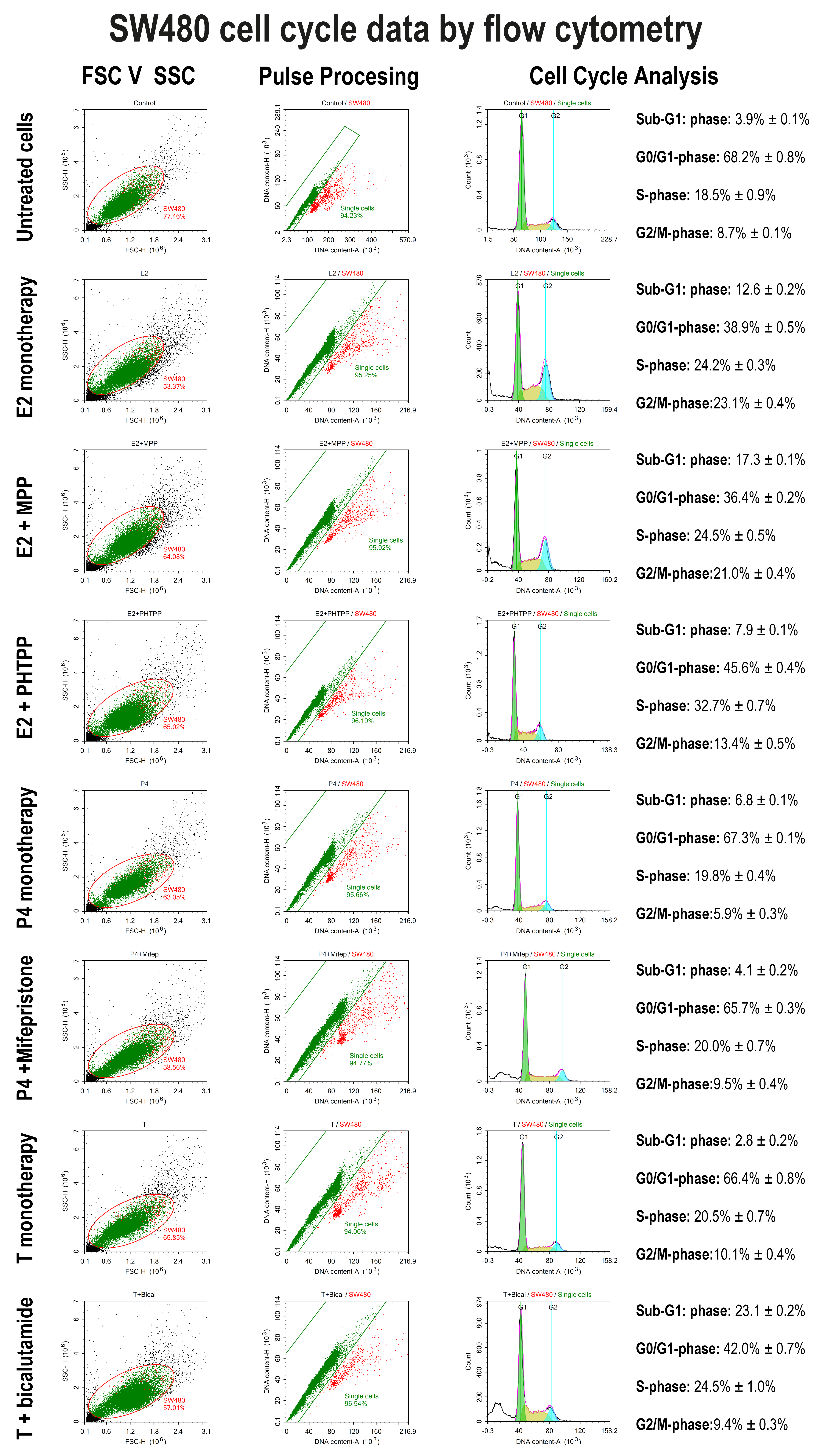

Supplement: Supplementary Figure 5 — Cell cycle analysis data for SW480 cells with the gating strategy used for each treatment group. The proportion of each phase of the cell cycle was determined for 20,000 single cell events using the NovoExpress cell cycle algorithm (right panel; histogram), and first gated on the SW480 cell population using forward scatter (FSC) vs side scatter (SSC) scatter plots (left panels), and then using DNA content height (H) vs. Area (A) scatter plots (middle panels) to calculate single cell events (pulse processing). The plots shown are representative of one of three similar experiments, and the percentage of each cell cycle phase is shown (mean ± SD; n = 3). [file Image_5.tif]

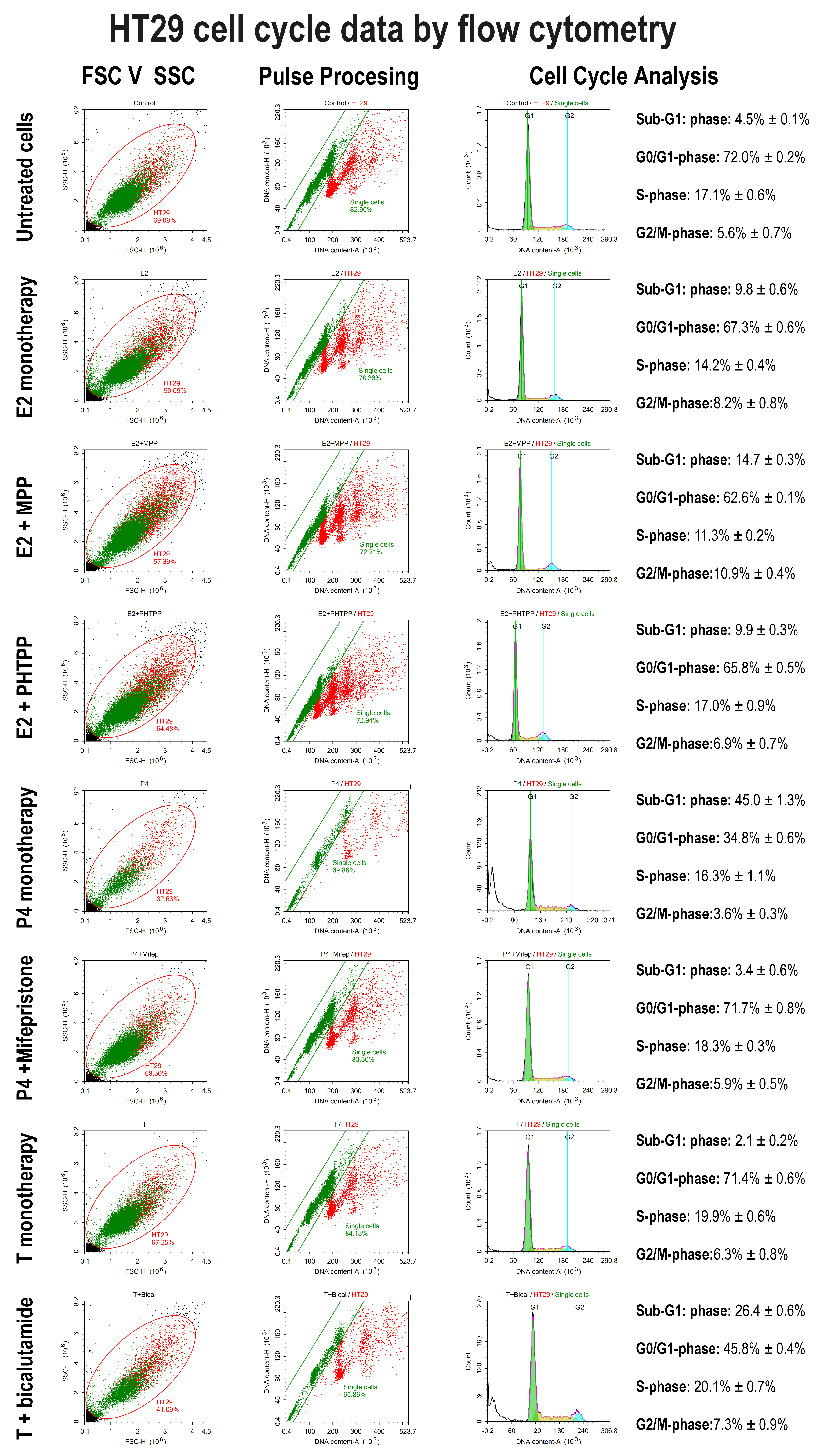

Supplement: Supplementary Figure 6 — Cell cycle analysis data for HT29 cells with the gating strategy used for each treatment group. The proportion of each phase of the cell cycle was determined for 20,000 single cell events using the NovoExpress cell cycle algorithm (right panel; histogram), and first gated on the HT29 cell population using forward scatter (FSC) vs side scatter (SSC) scatter plots (left panels), and then using DNA content height (H) vs. Area (A) scatter plots (middle panels) to calculate single cell events (pulse processing). The plots shown are representative of one of three similar experiments, and the percentage of each cell cycle phase is shown (mean ± SD; n = 3). [file Image_6.tif]
