# Supplementary material for: Profiling estrogen, progesterone, and androgen receptors in colorectal cancer in relation to gender, menopausal status, clinical stage, and tumour sidedness
Source: Front Endocrinol (Lausanne). 2023 May 3;14:1187259. doi: 10.3389/fendo.2023.1187259 (PMC10190606; doi:10.3389/fendo.2023.1187259)
Supplement: Supplementary file 7 [file Table_1.docx]

**Supplementary Table 1:** The clinicopathological characteristics according to CRC tumour side.

|  | ***Right-sided tumours***  ***(n = 41; 34.2%)*** | ***Left-sided tumours***  ***(n = 79; 65.8%)*** | ***P-value*** |
| --- | --- | --- | --- |
| ***Mean ± SD of Age (year)*** | 56 ± 14.4 | 58.8 ± 12.5 | 0.7 |
| ***Gender***  Male  Female | 21 (17.5%)  20 (16.7%) | 43 (35.8%)  36 (30%) | 0.7 |
| ***Tumour infiltration (T stage)***  T1  T2  T3  T4 | 0 (0%)  1 (0.8%)  25 (20.9%)  15 (12.5%) | 2 (1.6%)  12 (10%)  47 (39.2%)  18 (15%) | 0.07 |
| ***Median (IQR) of tumour volume (cm^3^)*** | 10.5 (5.0 – 18.5) | 9 (4.5 – 22) | 0.8 |
| ***Regional lymph node (N stage)***  N0  N1  N2 | 19 (15.8%)  10 (8.4%)  12 (10%) | 38 (31.7%)  28 (23.3%)  13 (10.8%) | 0.2 |
| ***Distant metastasis (M stage)***  M0  M1 | 30 (21.7%)  11 (9.2%) | 74 (61.6%)  5 (4.2%) | **0.002** |
| ***Histology***  Adenocarcinoma  Mucinous carcinoma | 26 (21.7%)  15 (12.5%) | 66 (55%)  13 (10.8%) | **0.01** |
| ***Differentiation***  Poor  Moderate  Well | 13 (10.8%)  24 (20%)  4 (3.4%) | 7 (5.8%)  48 (40%)  24 (20%) | **0.001** |
| ***Lymphovascular invasion***  No  Yes | 27 (22.5%)  14 (11.7%) | 51 (42.5%)  28 (23.3%) | 0.8 |
| ***Perineural invasion***  No  Yes | 32 (26.7%)  9 (7.5%) | 60 (50%)  19 (15.8%) | 0.8 |
| ***AJCC TNM stages***  Stages I/II (early)  Stages III/IV (advanced) | 13 (10.8%)  28 (23.3%) | 37 (30.8%)  42 (35%) | 0.1 |
